# Frictional behaviour of steel–steel and steel–concrete contact surfaces for offshore slip joint applications

**DOI:** 10.1038/s41598-026-65241-w

**Published:** 2026-08-05

**Authors:** Linus Joachim, Wenzhuo Ma, Vincent Oettel

**Affiliations:** Institute of Building Materials, Concrete Construction and Fire Safety (iBMB), Division of Concrete Construction, Technische Universität Braunschweig, Beethovenstraße 52, 38106 Braunschweig, Germany

**Keywords:** Offshore substructure, Slip joints, Push out tests, Fitting accuracy, Friction coefficient, Numerical simulation, Engineering, Materials science

## Abstract

As the energy sector is the main source of anthropogenic greenhouse gas emissions and energy demand is constantly rising, expanding renewable energy sources is imperative. Offshore wind energy is key to this expansion. In the future, more powerful offshore wind farms will be installed in deeper waters. However, this presents new technical and logistical challenges with regard to large-scale substructures. Modular jacket substructures are one possible solution for scalable offshore infrastructure. These require connection systems that are both structurally and technically efficient. Slip joints fulfil these requirements particularly by transferring loads through controlled friction at contacting surfaces rather than through permanent mechanical connectors. This study aims to develop an innovative modular jacket substructure incorporating steel–steel and steel–concrete slip joints. The article focuses on characterising interface behaviour, friction mechanisms, and load-bearing performance across four slip joint material combinations: rolled steel–rolled steel, ground steel–ground steel, rolled steel–concrete, and ground steel–concrete. Surface roughness was quantified using both tactile profilometry and high-resolution 3D scanning, while joint fitting accuracy was assessed exclusively through 3D scan-based geometric analysis. Experimental push-out tests were conducted to determine friction coefficients and load-bearing capacities for each configuration. Additionally, a finite element model was developed to simulate the push-out test and predict the load-bearing capacity. The results revealed that the rolled steel surface exhibited pronounced height fluctuations, irregularity, and distinct macro-waviness. These features promoted mechanical interlocking at the rolled steel–rolled steel interfaces, which in turn led to increasing load resistance and friction coefficients with progressing displacement. In contrast, the other surface combinations exhibited the typical transition from static to kinetic friction. However, the statistical evaluation did not identify significant differences in load-bearing capacity between the material combinations under identical normal stress, and only the rolled steel–rolled steel interface showed a friction coefficient dependent on the applied normal stress. The numerical model captured the observed shear-lag behaviour and reproduced the experimental response with good accuracy. The overall predictive performance of the FE model was demonstrated by a coefficient of determination (R^2^) of 0.87, a mean absolute percentage error (MAPE) of 16.57%, and a root mean square error (RMSE) of 17.02. Overall, the ground steel–concrete interface proved the most favourable configuration for slip joint applications, as it provided a balanced combination of frictional resistance, stable sliding behaviour, moderate installation forces, and uniform wear characteristics. These features may facilitate assembly and disassembly while maintaining reliable load transfer, making this configuration a promising option for offshore slip joint connections.

## Introduction

The global energy sector is responsible for roughly two-thirds of anthropogenic greenhouse gas emissions, making the reduction of fossil fuel dependency a central challenge of the coming decades. At the same time, primary energy demand is expected to rise by nearly one-third by 2040 due to continued industrialisation and population growth^1^. A significant and rapid expansion of renewable energy sources is therefore essential. Offshore wind energy is a key contributor in this transition: in the North Sea alone, the installed capacity is planned to increase from the current 30 GW to 120 GW by 2030 and to 300 GW by 2050^2,3^, with similarly ambitious expansion targets across the world^4,5^. Future offshore wind turbines will not only be installed in larger numbers but will also increase substantially in rated power and hub height. These offshore megastructures, exceeding 20 MW capacity^6^, must operate in deeper waters (> 50 m)^7–9^ and require correspondingly larger substructures with very high mass and geometric dimensions. The towing and installation of monolithic support structures with heights above 50 m is becoming increasingly difficult, costly and risky^10,11^.

A promising approach to address these challenges is the development of mega-substructures in the form of modular jacket substructures. Prefabricating slender and resource-efficient modules made of steel or high-performance concrete (HPC) under controlled factory conditions allows for: improved quality assurance, reduced transportation effort, lower offshore crane capacities, and faster, weather-independent installation^12–17^. These advantages make modular systems highly attractive. However, their feasibility depends critically on the availability of simple, robust, and durable offshore connection technologies that allow fast assembly and reliable load transfer under demanding environmental conditions.

**Fig. 1:**
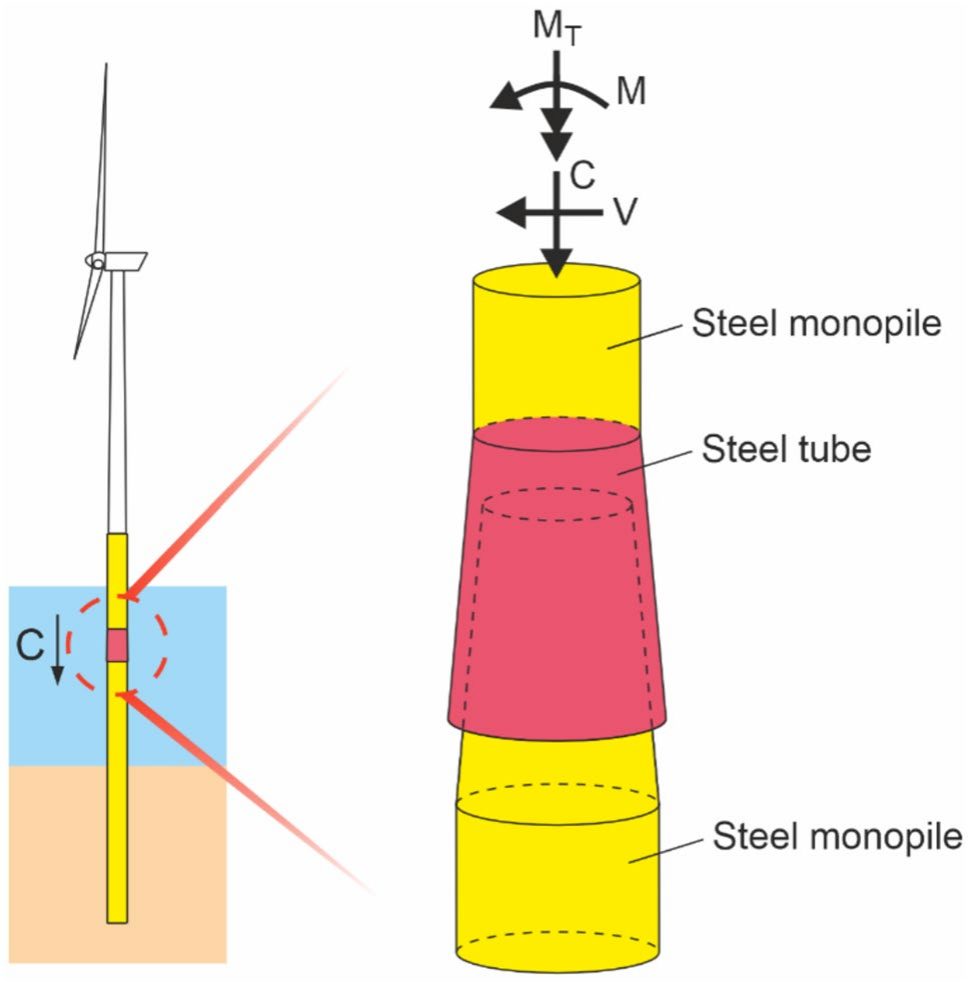
Monopile substructure made of steel with a slip joint connection (left, “C” denotes compressive load) and detail of the steel–steel slip joint connection (right).

Conventional offshore joints—bolted, welded, or grouted connections—are time-consuming, weather-dependent, cost-intensive, and often display high scatter in execution quality^18–20^. Additionally, welded and grouted joints are generally limited to regions above the waterline^18^, and are only partially dismantlable. Recent studies have investigated various advanced bolted and welded connection systems, such as moment-resisting joints^21^ and hybrid tubular joints^22,23^, which have demonstrated improved structural performance and enhanced fatigue life. Among these emerging connection technologies, slip joints (Fig. 1) represent a particularly attractive solution for offshore applications due to their simple assembly and friction-based load-transfer mechanism. A slip joint consists of two overlapping, tapered tubular segments that transfer axial forces and bending moments through friction alone. Slip joints require no bolts, no welding, no grout, minimal maintenance and can be installed above or below water. They can also be easily disassembled if the substructure needs to be demolished at the end of its planned service life^16,24^. Successful offshore applications on monopiles, such as the Borssele V wind farm, demonstrate the practical feasibility of steel–steel slip joints^25^.

Friction governs the load transfer mechanism of slip joints and is commonly approximated by the Coulomb law^26^. Reported friction coefficients vary widely depending on material combination, surface preparation, deformation mechanisms, and environmental conditions. For untreated steel–steel interfaces, friction coefficients typically range from 0.50 to 0.60, while coated surfaces exhibit lower values, between 0.05 and 0.42^27,28^. Investigations reported by Pijpers and Slot^28^ further indicate that environmental conditions, specifically dry air and seawater, have only a minor influence on the coefficient of friction; in both cases, corroded steel exhibited friction coefficients ranging from 0.42 to 0.75^25^. Concrete–concrete interfaces generally exhibit friction coefficients between 0.50 and 0.90, with ground concrete typically showing higher values than formwork-finished surfaces. Significant scatter is observed particularly at low normal stresses, and while friction tends to increase with normal stress, the data for higher pressures remain limited^29,30^. Design recommendations in Eurocode 2 specify values of 0.50 for surfaces cast against steel formwork and 0.60 for trowelled surfaces^31^, while Model Code 2010 provides values between 0.50 and 0.70^32^. For keyed dry joints that mobilise mechanical interlocking, values between 0.60 and 0.90 are reported for their smooth areas (e. g.^33^). In contrast, friction data for steel–concrete interfaces are relatively scarce. Studies on bond–slip behaviour between reinforcing steel and concrete suggest friction coefficients between 0.26 and 0.47^34,35^, while dry rolled steel–concrete interfaces have been reported to exhibit values around 0.57^35^. In addition to the friction coefficient itself, the load-transfer mechanism at steel–concrete interfaces is influenced by several interacting mechanisms, including mechanical interlocking arising from surface roughness, and friction mobilised by normal confinement. Previous push-out study^36^ has shown that stainless steel interfaces generally exhibit lower bond resistance than conventional carbon steel interfaces because of their smoother surface characteristics, whereas the incorporation of ultra-high-performance concrete can enhance interfacial resistance through fibre bridging and local mechanical interlocking effects. Consequently, the friction coefficient alone may not fully describe the interface behaviour, and the measured response often reflects the combined contribution of friction, surface morphology, confinement pressure, and material properties.

**Fig. 2:**
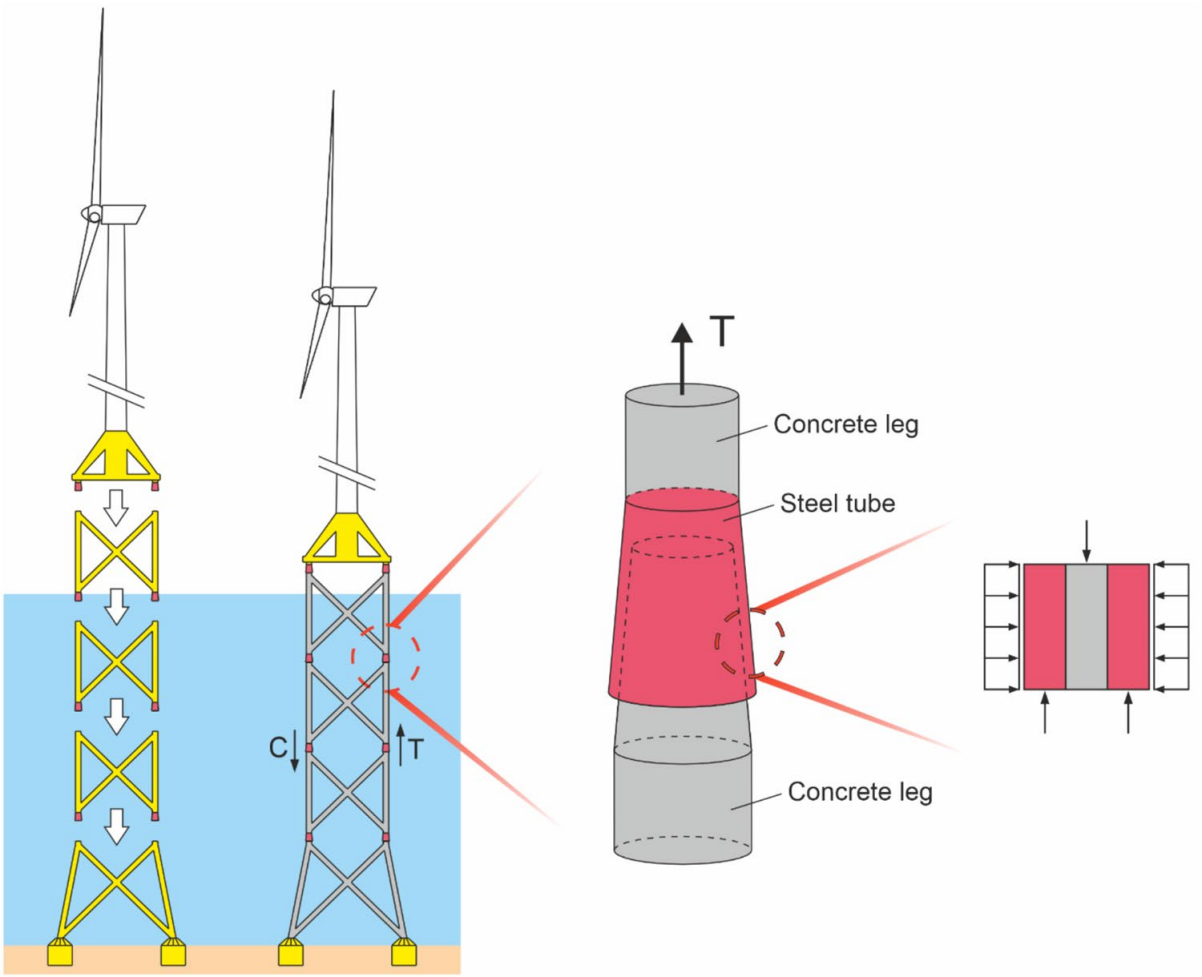
Conceptual illustration of steel and concrete modular mega-substructure with slip joint connections (left, “C” denotes compressive load, whereas “T” denotes tensile load), detail of the steel–concrete slip joint connection (middle), and the representative interface segment investigated in this study (right).

Although slip joint technology is considered highly promising for offshore substructures, existing research and applications focus almost exclusively on steel–steel slip joints for monopiles substructures (e.g.^18–20,24,25^). However, when considering long-term durability, corrosion risk, and overall lifecycle cost, a steel–concrete slip joint could offer significant advantages. Despite this potential, no systematic research currently exists on steel–concrete slip joints. In particular, there is a complete lack of experimental, numerical, and analytical studies that address the interface behaviour, friction mechanisms, material compatibility, or environmental effects relevant to offshore conditions. This absence of knowledge represents a critical research gap that limits the development of cost-effective and durable slip joint solutions for the offshore industry. Additionally, as offshore megastructures are intended to be installed in deeper waters, while conventional monopile substructures remain more suitable for shallow sites^37,38^, the concept of developing modular jacket substructures composed of multiple modules connected by slip joints has emerged. However, slip joints have so far been limited to monopile substructures, with no studies addressing their application in modular jacket structures. While in monopiles the slip joint is permanently overloaded by the self-weight of the tower and turbine (Fig. 1), jacket substructures experience alternating axial force pairs from environmental and operational loading (wind, waves, turbine operation, etc.), resulting in slip joints being subjected to both compression and tension (Fig. 2, left). Therefore, this study aims to develop an innovative modular jacket substructure incorporating steel–steel and steel–concrete slip joints under compression but, more importantly, under tension, as shown in Fig. 2 (left and middle). To establish a fundamental understanding of the steel–steel and steel–concrete slip joint behaviour, a representative local interface segment of the joint (Fig. 2, right) was isolated and examined experimentally and numerically to characterise the underlying friction mechanisms at the contact surfaces. Specifically, the objectives of this study are to:

- investigate the frictional behaviour and load-bearing response of steel–steel and steel–concrete slip-joint interfaces;
- characterise the surface roughness and fitting accuracy of the contact interfaces using high-resolution 3D scanning;
- develop and validate a finite element model for predicting the frictional and load-transfer behaviour of slip joints.

This study provides the first systematic comparison of frictional behaviour in steel–steel and steel–concrete slip-joint interfaces, combining surface characterisation, friction testing, and numerical modelling. The findings establish fundamental relationships between surface roughness, interface fitting accuracy, and frictional performance, while demonstrating the potential of steel–concrete interfaces as an alternative to conventional steel–steel configurations. From a lifecycle perspective, including durability, corrosion mitigation, and associated costs, steel–concrete slip joints warrant further investigation for application in modular offshore structures.

## Experimental program

### Sample fabrication and testing matrix

**Fig. 3:**
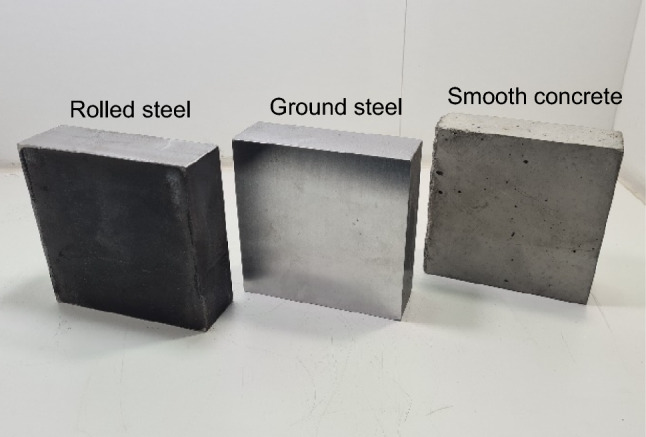
Steel and concrete prisms.

The steel prisms (height/width/thickness = 15/15/5 cm) were fabricated by precision milling from rolled and cold-drawn S355 steel. The prism dimensions were selected to ensure a representative contact area while remaining compatible with the available testing equipment and loading capacity. Furthermore, the selected geometry is consistent with specimen dimensions used in previous investigations of frictional interfaces^39^. The surfaces of the prisms produced from rolled steel were left in their as-milled condition, whereas those made from cold-drawn steel were ground to achieve a specified surface roughness of ${R}_{\mathrm{a}}$ ≤ 0.8 μm. Both types of steel prisms are shown in Fig. 3.

The concrete prisms, as also shown in Fig. 3, were cast in cube formwork (height/width/thickness = 15/15/15 cm) against ground steel prisms to produce high-strength concrete specimens of grade C80/95 with final dimensions of 15/15/5 cm. The concrete mixing followed the composition used by Basaldella et al.^40^, with a water-to-cement ratio of 0.35. The superplasticiser was added at 1.0 wt.% of the cement content, and the stabiliser at 0.57 wt.%. Table 1 presents the mixing proportion. After casting, the samples were covered with foil, demoulded after 24h. They were subsequently cured under foil for six days and then stored at room temperature until testing. To characterise the compressive properties of hardened concrete, cylinders with dimensions diameter/height = 10/20 cm were cast and subjected to the same curing and storage conditions as the prisms. The concrete compressive strength *f*_cm_, E-modulus *E*_cm_, and bulk density *ρ*_cm_ were determined at the time (55 days after casting) of the push-out tests and are reported in Table 2.

**Table 1:** Concrete mixing composition.

Component	Quantity [kg/m^3^]
Cement: Holcim Sulfo 5R, CEM I 52.5 R-SR3(na)	500
Fine sand: Quarzwerke H33	75
Sand 0/2	850
Basalt 2/5	350
Basalt 5/8	570
Superplasticiser: BASF MasterGlenium® ACE 460	5.00
Stabiliser: BASF MasterMatrix® SDC 100	2.85
Water	176

**Table 2:** Compressive and material properties of concrete.

*f*_cm_ [N/mm^2^]	*E*_cm_ [N/mm^2^]	*ρ*_cm_ [kg/m^3^]
89.7	39,062.0	2,463.7

As presented in Table 3, four different material combinations were proposed for the joint: rolled steel–rolled steel (S1), ground steel–ground steel (S2), rolled steel–concrete (S3), and ground steel–concrete (S4). In addition to determining the surface roughness of the three materials—rolled steel, ground steel, and concrete—the joint-fitting accuracy was evaluated, and push-out tests were performed for all four joint configurations under three levels of normal stress: 1, 5, and 10 N/mm^2^, with each test conducted three times. The normal stress levels were selected to represent the range of interface pressures expected in practical slip-joint applications. They correspond to the contact pressures resulting from the circumferential tensile stresses typically occurring in slip-joint connections and are consistent with values reported in the literature^41^. The corresponding testing procedures are described in Sect. “Test methods”.

**Table 3:** Sample matrix.

Series	Sample name	Side prisms	Central prism	Normal stress [N/mm^2^]
S1	S1-1	Rolled steel	Rolled steel	1
	S1-5			5
	S1-10			10
S2	S2-1	Ground steel	Ground steel	1
	S2-5			5
	S2-10			10
S3	S3-1	Rolled steel	Concrete	1
	S3-5			5
	S3-10			10
S4	S4-1	Ground steel	Concrete	1
	S4-5			5
	S4-10			10

### Test methods

#### Tactile profilometry

**Fig. 4:**
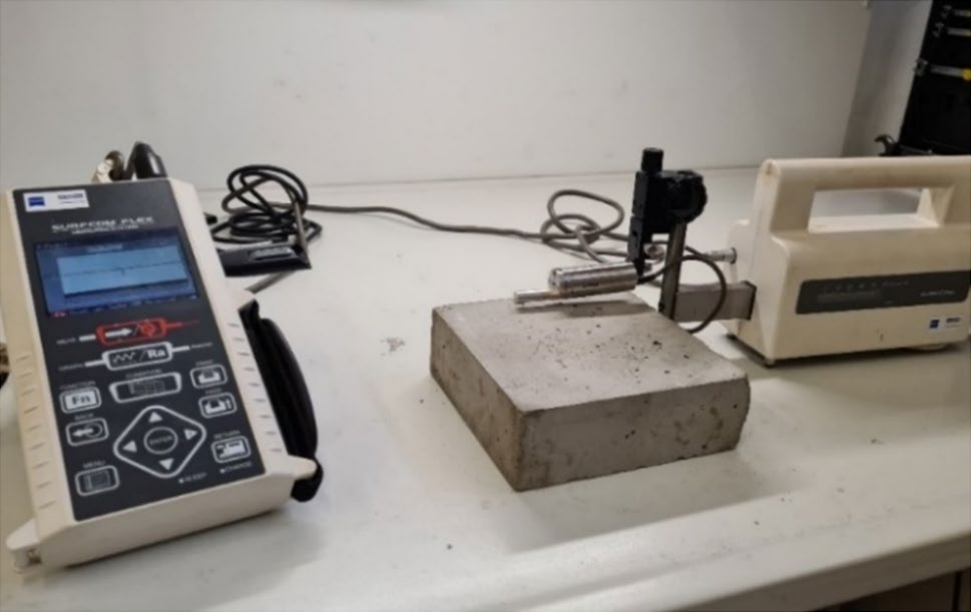
Measurement of the joint surface using tactile profilometry.

The roughness of the three types of prisms was determined by means of tactile profilometry, as shown in Fig. 4. With a diamond stylus tip moving across the surface at a constant speed, the surface measurement profile results from the vertical displacement of the stylus tip.

**Fig. 5:**
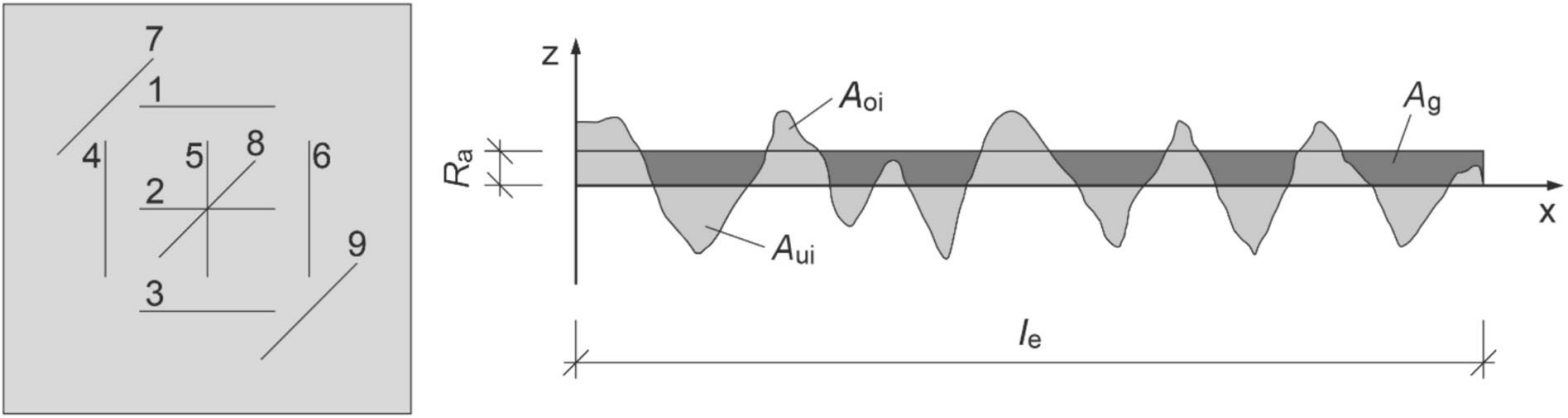
Top view of the joint surface with scanning directions for the tactile roughness measurements (left) and representation of the arithmetic mean height ${R}_{a}$ according to^42^ as a section (right).

To obtain reliable roughness measurements for each surface, three profilometer measurements were performed in each of three directions—horizontal (1, 2 and 3 in Fig. 5, left), vertical (4, 5 and 6 in Fig. 5, left) and diagonal (7, 8 and 9 in Fig. 5, left)—resulting in nine measurements per surface. The measurement length for each scan was 5 cm, as specified by the profilometer device. Each measurement was acquired once. The nine measurements per surface were used to obtain a representative roughness characterisation and to account for potential anisotropy of the surface texture.

The primary parameter evaluated was the arithmetic mean height ${R}_{\mathrm{a}}$, which represents the mean of the absolute profile deviations from the centerline over the evaluation length ${l}_{\mathrm{e}}$^43^, as illustrated in Fig. 5, right. According to ISO 21920–2^43^, ${R}_{\mathrm{a}}$ is defined by Eq. (1):

1 $${R}_{\mathrm{a}}=\frac{1}{{l}_{\mathrm{e}}}\underset{0}{\overset{{l}_{\mathrm{e}}}{\int }}\left|z(x)\right|dx$$

In practical terms, ${R}_{\mathrm{a}}$ is calculated by summing all areas above (${A}_{\mathrm{o}\mathrm{i}}$) and below (${A}_{\mathrm{u}\mathrm{i}}$) the profile centerline, forming the total area (${A}_{\mathrm{g}})$, and dividing by the evaluation length (${l}_{\mathrm{e}})$, as shown in Eq. (2):

2 $${R}_{\mathrm{a}}=\frac{1}{{l}_{\mathrm{e}}}\underset{0}{\overset{{l}_{\mathrm{e}}}{\int }}\left|z(x)\right|dx=\frac{\sum {A}_{\mathrm{o}\mathrm{i}}+\sum {A}_{\mathrm{u}\mathrm{i}}}{{l}_{\mathrm{e}}}=\frac{{A}_{\mathrm{g}}}{{l}_{\mathrm{e}}}$$

For a balanced surface profile, the areas above and below the centerline are equal, as shown in Eq. (3):

3 $$\sum {A}_{\mathrm{o}\mathrm{i}}=\sum {A}_{\mathrm{u}\mathrm{i}}$$

#### High-resolution 3D scanning

**Fig. 6.**
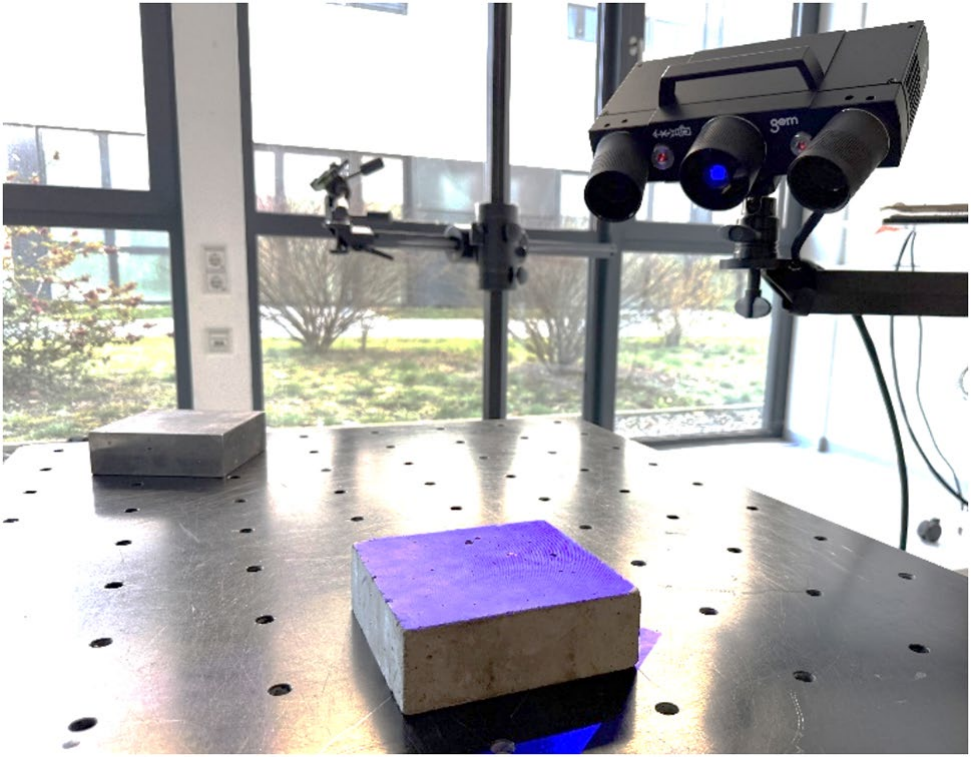
3D scan of the joint surface with the structured-light 3D scanner ATOS Q.

In addition to tactile profilometry, an innovative approach using high-resolution 3D scanner of the type ATOS Q (structured-light 3D scanner) was applied to digitise the surface topography (Fig. 6). The scanner provides up to 12 million measuring points per scan, enabling high-resolution surface acquisition. Prior to the measurements, the scanner was calibrated using the manufacturer’s calibration panel in accordance with the standard measurement procedure.

Since ZEISS Inspect, the evaluation software associated with the scanner, did not previously offer the option of determine roughness parameters from the scan data, an add-on for ZEISS Inspect was developed in cooperation with Carl Zeiss GOM Metrology GmbH as part of a DFG initial funding project within CRC 1463 “Integrated Design and Operational Methodology for Offshore Megastructures” (see also^42,44^). This approach enables the automated calculation of roughness parameters, such as the arithmetic mean height ${S}_{\mathrm{a}}$ of a surface over a given area, according to the following Eq. (4) ref.^45^:

4 $${S}_{\mathrm{a}}=\frac{1}{A}\underset{\widetilde{A}}{\overset{}{\iint }}\left|z(x,y)\right|dxdy$$

where *A* is the evaluation area, and *z(x,y)* is the ordinate value of the surface profile. To determine ${S}_{\mathrm{a}}$ from the scan data, the individual scan points are converted into a triangle-based surface model using Delaunay triangulation. For each evaluation point, a Gaussian regression surface fit is computed within a defined neighbourhood. The fitting plane is iteratively shifted until the volumes located above and below the plane are equal. The sum of the absolute volumes is then normalised by the base area, yielding the arithmetic mean height ${S}_{\mathrm{a}}$. This procedure is repeated for all evaluation points, allowing ${S}_{\mathrm{a}}$ values to be obtained for the entire scanned surface and visualised, for example, as scalar plots. A supplementary script enables statistical post-processing of the roughness data, including the computation of all relevant statistical descriptors. The scan method evaluates the complete contact surface and thus also provides information on macro-roughness. In this study, the term macro-roughness refers to the overall waviness of the joint surface, whereas micro-roughness refers to the small-scale surface texture.

When comparing the ${S}_{\mathrm{a}}$ parameter (Eq. (4)) determined by scanning with the ${R}_{\mathrm{a}}$ parameter (Eq. (1)) determined by tactile measurement, it must be taken into account that both represent the arithmetic mean height; however, ${S}_{\mathrm{a}}$ is evaluated over an area (3D), whereas ${R}_{\mathrm{a}}$ is determined along a profile (2D). Although the underlying evaluation concepts are comparable, the two parameters are not directly equivalent from a measurement standpoint. To facilitate interpretation of the roughness values obtained from the scanning method, ${S}_{\mathrm{a}}$ is therefore assumed to be approximately equal to the mean ${R}_{\mathrm{a}}$ value for each surface (see also^42^). In accordance with the relevant standards^43,45^, they are designated using different symbols.

**Fig. 7:**
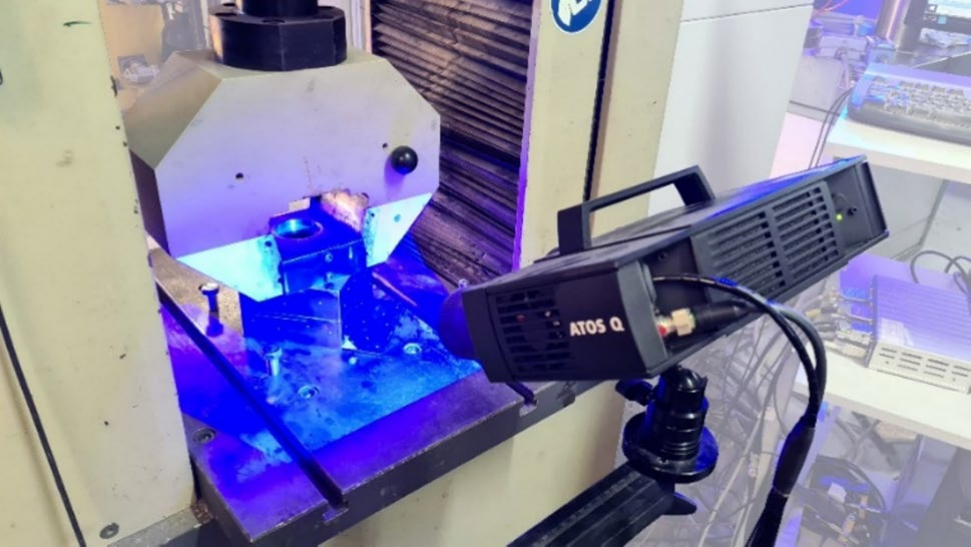
Evaluation of fitting accuracy between two prisms using 3D scanning.

Beyond characterizing surface roughness, the 3D scanner was employed to quantify the real contact area and the surface distance resulting from the roughness, thereby enabling an evaluation of the joint fitting accuracy. The assessment was carried out in two steps. First, the two friction partners were assembled and scanned together in a testing machine while applying different joint normal forces *N* perpendicular to the interface (as shown in Fig. 7). Three load levels, i.e., 22.5, 112.5, and 225.0 kN, were selected, corresponding to nominal joint normal stresses of 1 N/mm^2^, 5 N/mm^2^ and 10 N/mm^2^ (see Table 3), respectively, based on the apparent contact area (height/width = 15/15 cm) (see also^42^). This variation in load made it possible to examine the influence of the prestressing level on the resulting surface gap. In the second step, each friction partner was scanned individually using the same high-resolution optical setup. The individual surface models were then digitally aligned and combined with the joint scans to determine the local separation between the surfaces. From these datasets, the surface gap ${a}_{\mathrm{j}}$ was computed, providing a quantitative measure of the fitting quality of the joints. Colour-mapped surface plots were generated to visualise the spatial distribution of the gap.

#### Push-out test

**Fig. 8:**
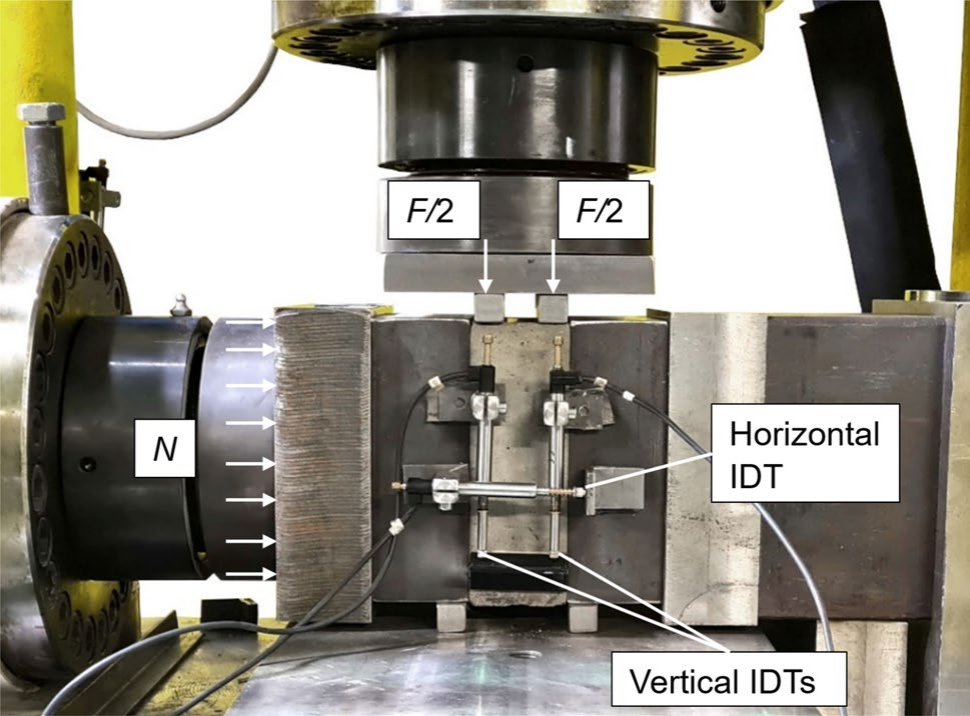
Setup of push-out test (illustrated using configuration S3 as an example).

The push-out tests were performed in accordance with EN 1994–1-1^46^. As shown in Fig. 8, the normal force *N* was applied using a horizontal hydraulic cylinder, while the push-out force *F* was introduced through a vertical hydraulic cylinder. Based on the target normal stresses ${\sigma}_{\mathrm{n}}$ of 1, 5 and 10 N/mm^2^ listed in Table 3, the corresponding normal forces *N* were set to 22.5, 112.5 and 225.0 kN, respectively, as calculated based on the apparent contact surface (height/width = 15/15 cm). The normal force *N* was maintained constant throughout each test. After applying *N*, the central prism was displaced downward at a constant rate of 0.01 mm/s (= *F/2*). The displacement rate of 0.01 mm/s was selected to ensure quasi-static loading conditions. Force and displacement data were recorded at a sampling frequency of 10 Hz. The relative vertical displacement between the central prism and the side prisms was monitored using two vertical inductive displacement transducers (IDTs). In addition, a horizontal IDT was used to measure the relative lateral displacement between the left and right-side prisms. Each test was terminated once a vertical displacement of 8 mm was reached, as a stable friction plateau had already been established and no further significant changes in the frictional response were observed. Three replicate tests were performed for each series. The load cells and displacement transducers used for the push-out tests were calibrated prior to testing. All tests were conducted under ambient laboratory conditions.

## Results and discussions

### Roughness

#### Roughness evaluated by tactile profilometry

**Fig. 9:**
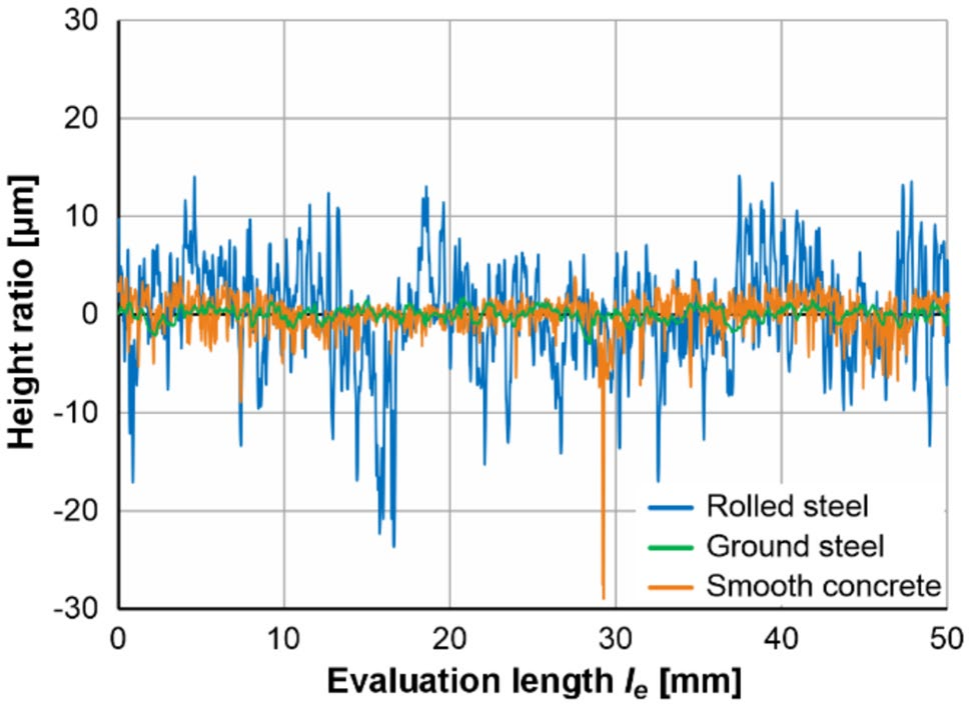
Representative height profile of the examined surfaces.

Figure 9 presents a representative height profile measured in the vertical direction, and Table 4 summarises the roughness parameter ${R}_{\mathrm{a}}$ obtained from tactile profilometry. The rolled steel surface exhibits pronounced height fluctuations and irregularity, reflecting a strong microstructural imprint from the hot-rolling process. In contrast, the ground steel profile is noticeably smoother and more uniform, characterised by a fine, periodic texture with low-amplitude peaks produced by grinding. The smooth concrete surface displays a comparable overall amplitude to ground steel but includes occasional deep pits, indicating the presence of localised surface defects. From an engineering perspective, the observed differences in surface topography indicate varying interface conditions, which may contribute to differences in the frictional behaviour of the investigated joints.

**Table 4:** Statistical summary of the surface roughness parameter ${R}_{a}$ obtained from tactile profilometry.

Surface	${R}_{\mathrm{a}}$ mean value [μm]	Standard deviation [μm]	Coefficient of variation [-]	5% Quantile value [μm]	95% Quantile value [μm]
Rolled steel	3.98	0.48	0.12	3.54	5.05
Ground steel	0.52	0.25	0.44	0.25	1.11
Concrete	1.02	0.99	0.66	0.60	5.30

**Fig. 10:**
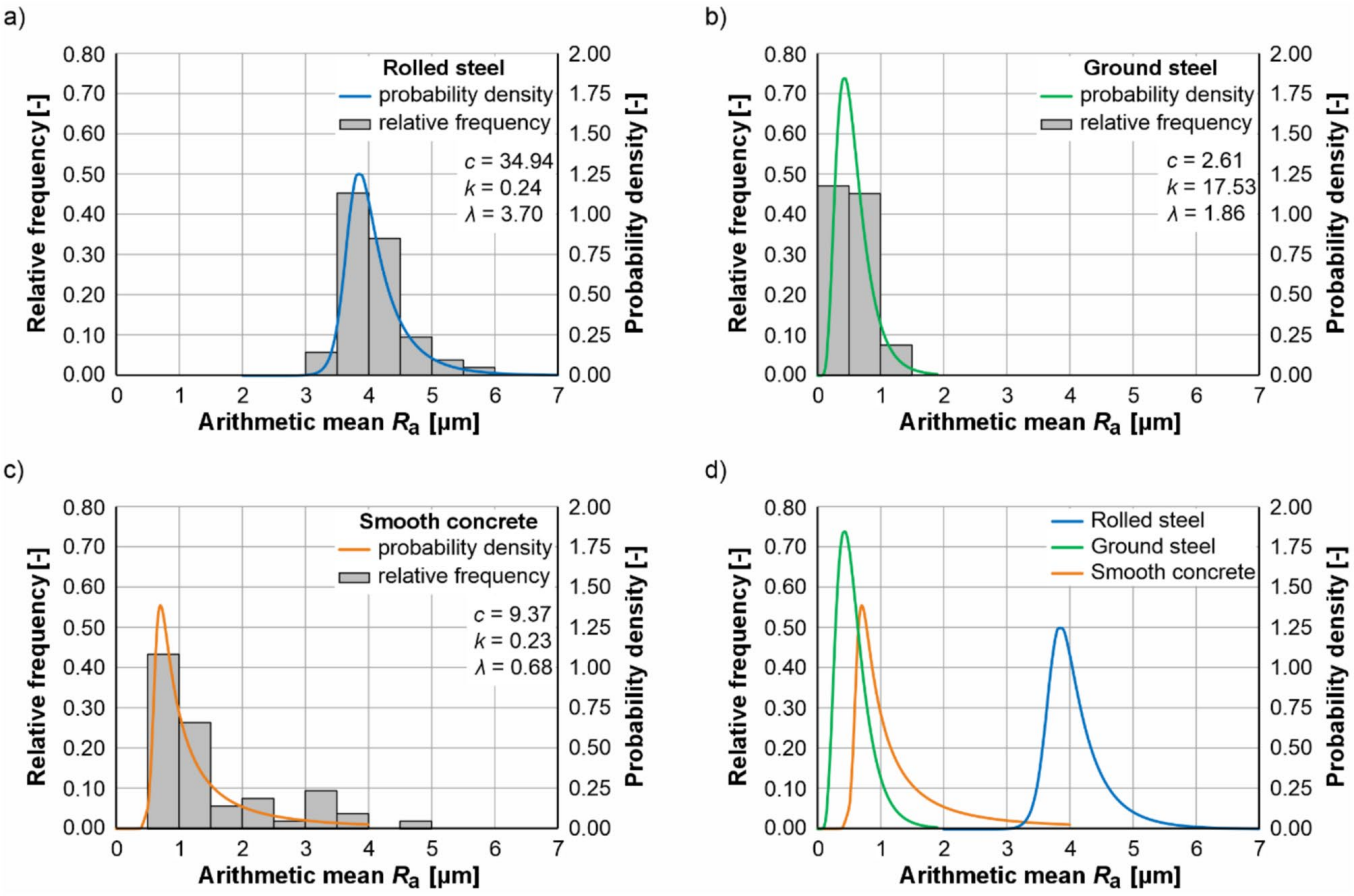
Distribution of the arithmetic mean height ${R}_{a}$ of: (**a**) rolled steel, (**b**) ground steel, (**c**) smooth concrete and (**d**) compilation of the investigated surfaces.

The distribution of the arithmetic mean height ${R}_{\mathrm{a}}$ is shown in Fig. 10 and is well described by a Burr type XII distribution^47^. The probability density function of the Burr (type XII) distribution is given in Eq. (5):

5 $$f\left(x|c,k,\lambda \right)=\frac{ck}{\lambda }{(\frac{x}{\lambda })}^{c-1}{\left[1+{(\frac{x}{\lambda })}^{c}\right]}^{-(k+1)}$$

where *c* is a shape parameter that governs the curvature near the origin; *k* is a tail-shape parameter that controls the decay of the right tail; *λ* is a scale parameter that sets the characteristic magnitude of *x*. As highlighted in Fig. 10, rolled steel exhibited a large *λ* combined with a small *k*, indicating a high overall roughness level with frequent extreme asperities originating from the hot-rolling process. Ground steel, in contrast, shows a much smaller *λ* and a largest *k*, reflecting a highly uniform surface with rapid tail decay. Smooth concrete displays the smallest characteristic roughness (λ = 0.68) but, like rolled steel, a very small k = 0.24, revealing that although its average roughness is low, the heterogeneous microstructure leads to occasional deep pits and a heavy right tail. To test whether the roughness of ground steel and smooth concrete is statistically different, a likehood-ratio test with null hypothesis H_0_: the ${R}_{\mathrm{a}}$ of ground steel and smooth concrete share the same Burr XII distribution was conducted. The likelihood-ratio statistic was *T* = 48.99, which follows a *χ*^*2*^ distribution with 3 degrees of freedom. The corresponding p-value (1.3 × 10^−10^) strongly rejects H_0_, indicating that the two groups exhibit statistically distinct roughness distributions. In practical terms, smooth concrete shows a markedly heavier-tailed roughness profile than ground steel, indicating more frequent occurrence of large asperities in addition to its higher average roughness level.

#### Roughness evaluated by structured-light 3D scanning

**Fig. 11:**
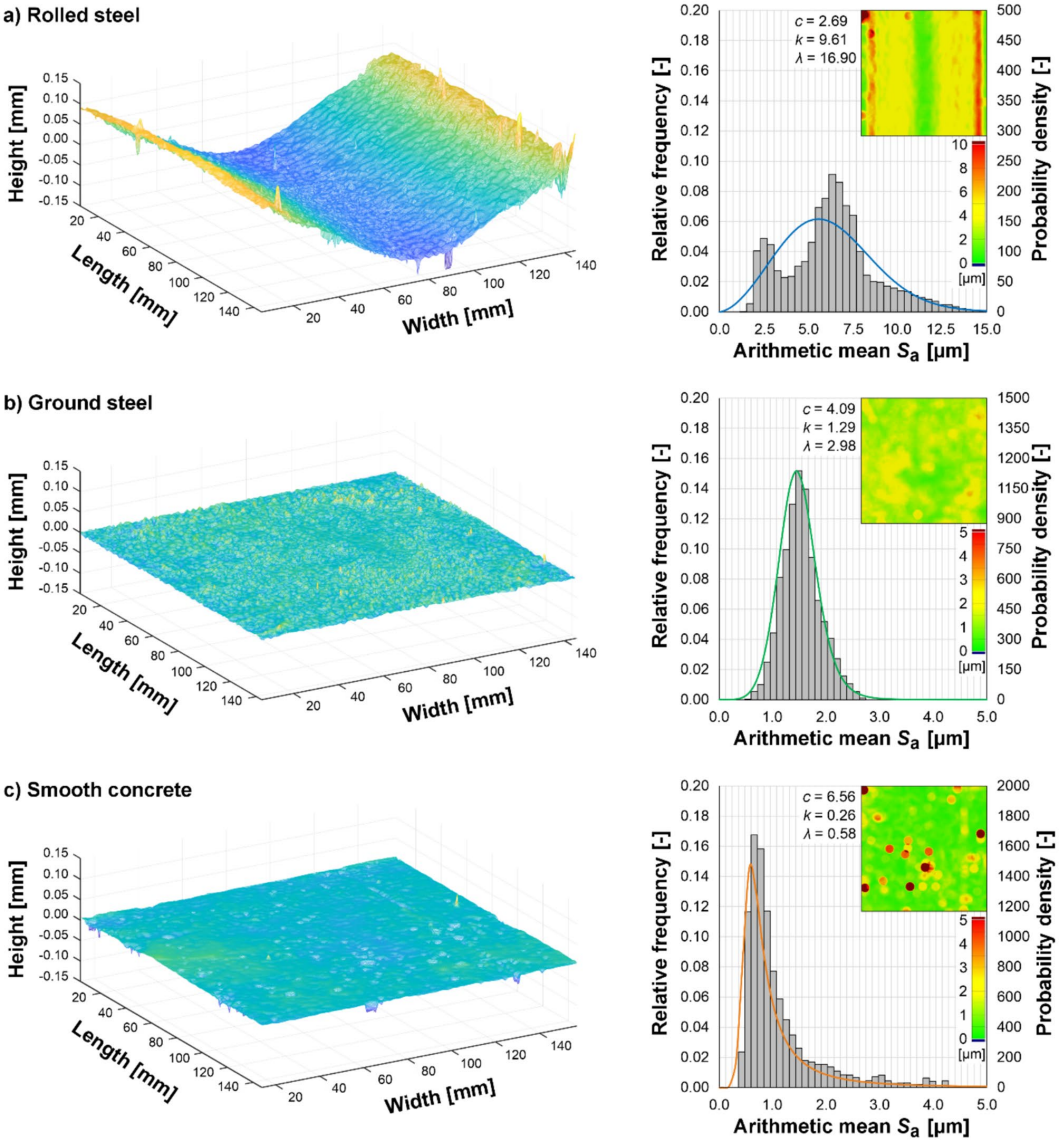
Isometric surface maps (left) and corresponding distributions of the arithmetic mean roughness ${S}_{a}$ with scalar probability density plots (right) determined from the 3D scan data for: (**a**) rolled steel, (**b**) ground steel and (**c**) smooth concrete.

Figure 11 shows the isometric surface maps and Burr type XII distributions (Eq. (5)) of the arithmetic mean roughness ${S}_{\mathrm{a}}$, together with the corresponding scalar plots, whereas Table 5 summarises the roughness parameter ${S}_{\mathrm{a}}$ obtained from 3D scanning. Consistent with the tactile profilometry results, the rolled steel exhibits the highest and most widely scattered ${S}_{\mathrm{a}}$ values. In contrast to the tactile measurements, which indicated a higher mean ${S}_{\mathrm{a}}$ for smooth concrete than for ground steel, the 3D scanning shows that smooth concrete has a slightly lower mean ${S}_{\mathrm{a}}$ than ground steel. Nevertheless, as shown in Fig. 11 the concrete surface again displays a markedly heavier-tailed roughness distribution, reflecting more frequent occurrence of large asperities. A likelihood-ratio test confirms that ground steel and smooth concrete follow fundamentally different roughness distributions (*T* = 5.47 × 10^5^, *df* = 3, p-value ≈ 0). Additionally, isometric surface maps illustrate that the rolled steel exhibits not only pronounced micro-roughness but also a noticeable macro-scale waviness, visible as a surface bulge (Fig. 11a) left). Besides the assessment of surface roughness, the full-surface scan enables the quantification of such macro-waviness and the fitting accuracy of mating surfaces, which is particularly relevant for the evaluation of complete slip-joint connections. The three-dimensional (exaggerated) representations further make the asperity patterns of all investigated surfaces clearly distinguishable.

**Table 5:** Statistical summary of the surface roughness parameter ${S}_{a}$ obtained from 3D scanning.

Surface	Mean value [μm]	Standard deviation [μm]	Coefficient of variation [-]	5% Quantile value [μm]	95% Quantile value [μm]
Rolled steel	5.92	2.58	0.42	2.23	10.65
Ground steel	1.47	0.37	0.25	0.90	2.13
Concrete	0.78	0.82	0.76	0.42	2.96

#### Comparison between the two methods

Both approaches revealed the notably high roughness of rolled steel, with the 3D scanning technique additionally capturing pronounced macro-scale waviness. Ground steel and smooth concrete exhibited comparable average roughness values in both measurement methods; however, smooth concrete features a higher frequency of large asperities, reflected in its heavier-tailed roughness distribution. Quantitatively, the mean roughness obtained by 3D scanning was approximately 49% higher for rolled steel (5.92 vs. 3.98 μm) and 183% higher for ground steel (1.47 vs. 0.52 μm), whereas smooth concrete yielded a 24% lower mean value (0.78 vs. 1.02 μm). Furthermore, the coefficient of variation differed between the methods, increasing from 0.12 to 0.42 for rolled steel, decreasing from 0.44 to 0.25 for ground steel, and remaining at a comparable level for concrete (0.66 and 0.76, respectively). These differences indicate that the full-field optical approach captures additional surface characteristics, particularly larger-scale surface undulations, whereas tactile profilometry primarily reflects local profile roughness along individual measurement paths. The proposed 3D scanning methodology offers several advantages over tactile profilometry, including:

- full-area acquisition and digitalisation of the entire interface surface;
- absence of limitations regarding the size of the surface to be analysed;
- automated, contact-free, and rapid evaluation of the complete roughness field;
- capability to capture both micro- and macro-scale roughness characteristics.

Although 3D scanning requires higher initial investment costs and setup effort, these disadvantages are offset by automated full-field acquisition, which reduces manual effort, enables reproducible analyses, and provides substantially more comprehensive information on the interface topography. Similar systems are also used, for example, in automotive and aircraft manufacturing.

### Joint fitting accuracy

The surface-gap maps in Table 6 provide a visual and quantitative assessment of the joint-fitting accuracy for the four surface configurations under increasing normal stresses ${\sigma}_{\mathrm{n}}$. Across all samples, a consistent reduction in the average surface gap $\overline{{a }_{\mathrm{j}}}$ is observed as the applied normal stress increases, indicating improved conformity between the mating surfaces. Due to strong macro-waviness as reported in Sect. “Roughness”, S1 shows the largest positive gaps. The colour distribution reveals persistent macro-scale waviness, which prevents full surface contact. Although increasing pressure slightly reduces the gap, the interface retains a notable degree of separation due to the inherent roughness and curvature of the surface. S2 exhibits the most effective fit among all configurations. The colour maps show a homogeneous distribution, indicating highly planar surfaces that achieve near-complete contact when loaded. This demonstrates that smooth ground steel surfaces accommodate joint pressure efficiently, resulting in excellent surface conformity. S4 behaves similarly to S3, though with smaller initial gaps. As pressure increases, the mean gap shifts from slightly positive to mildly negative, suggesting that the surface is more compliant and able to deform under load. However, the colour maps still show spatially distributed defects typical of concrete surfaces, constraining full-area contact. S3 shows moderate fitting performance. Although gaps decrease with increasing pressure, the surface retains noticeable regions with elevated separation, likely associated with the natural asperities and voids typical of concrete. The average gap remains positive, indicating that micro-defects and heterogeneity limit the achievable contact quality compared to S2. From an engineering perspective, the measured surface gaps provide an indication of the achievable contact quality between the mating surfaces. Smaller residual gaps correspond to a larger effective contact area and are therefore beneficial for efficient load transfer across the interface, whereas larger gaps indicate less contact between the joined surfaces.

**Table 6:**
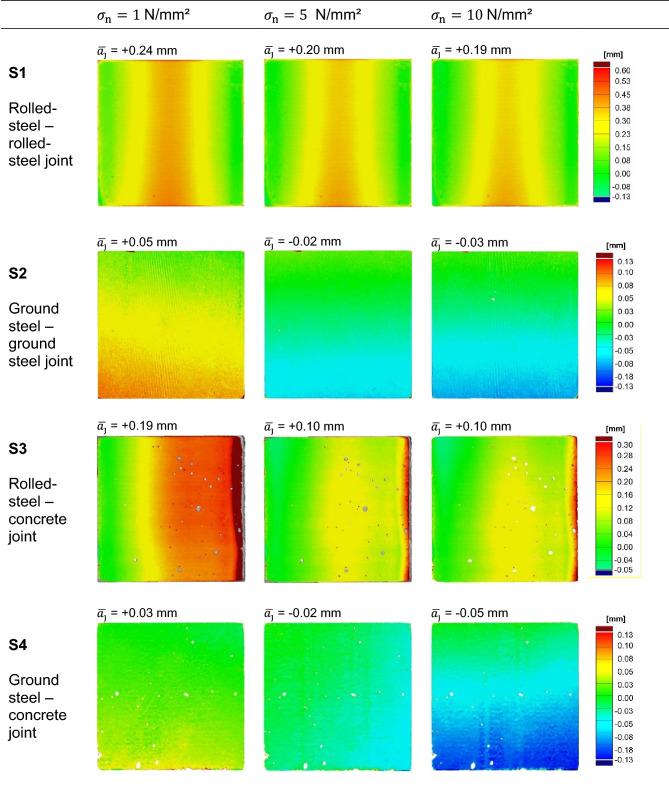
Surface-gap maps of different joint configurations S1 to S4 under varying normal stresses.

The results demonstrate that 3D structured-light scanning provides a highly effective means of evaluating joint-fitting accuracy. By capturing the full geometry of the contact surfaces, the scanner enables precise quantification of residual surface gaps and their spatial distribution across the entire interface. The method is particularly well suited for quality control during the fabrication of mechanical joints, such as slip joint connections, where reliable surface conformity is critical for effective load transfer and long-term structural performance. Additionally, since offshore wind turbines are subjected to cyclic loads, the influence of long-term wear or repeated loading on joint-fitting accuracy represents an important topic for future research and will be investigated in subsequent studies.

### Load-bearing behaviour and friction coefficient

#### Surface failure characterisation

**Fig. 12:**
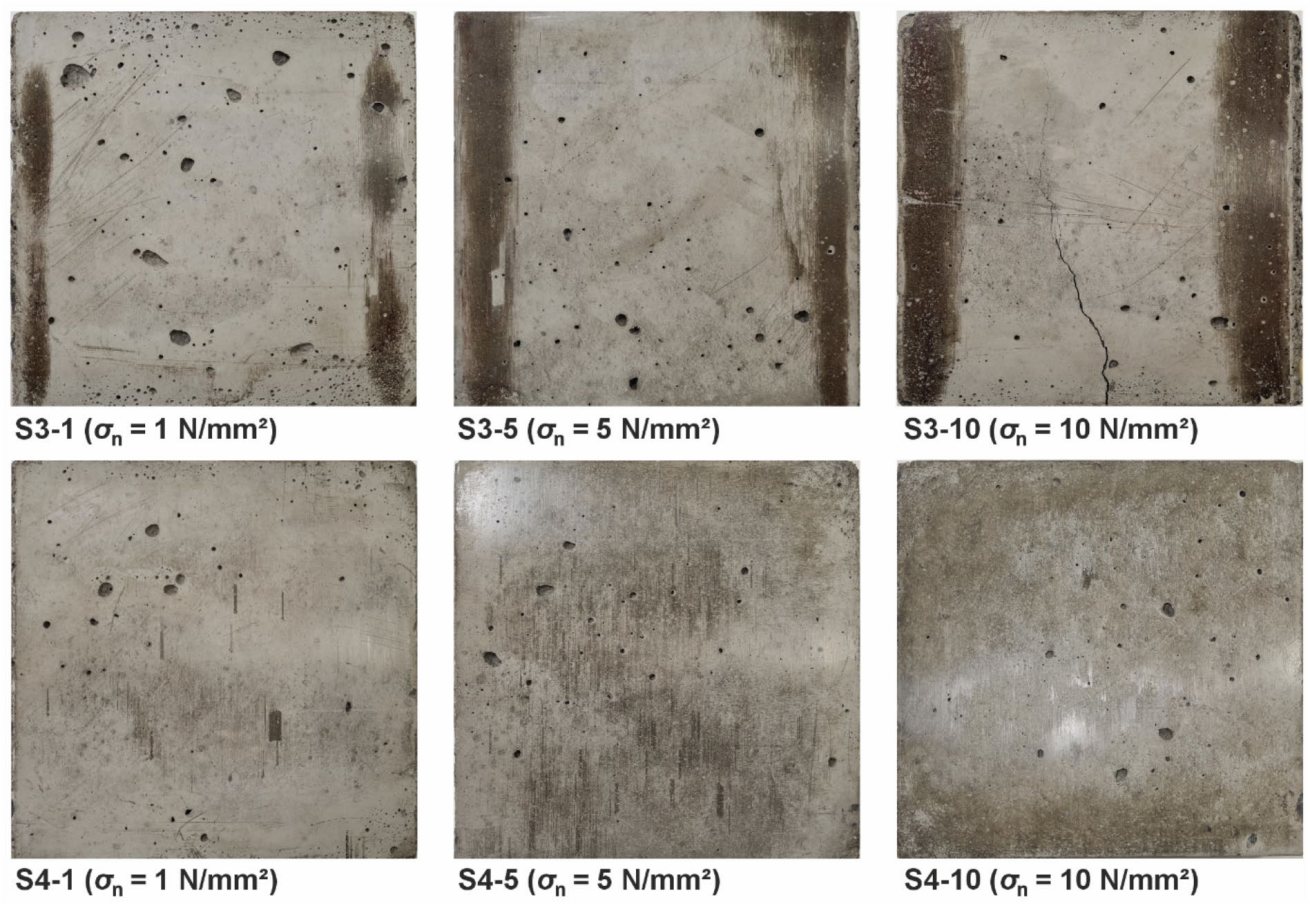
Post-test concrete surface damage for S3 (top) and S4 (bottom) depending on the normal stress ${\sigma}_{n}$.

All prisms remained structurally intact after the push-out tests, with the exception of the concrete prisms in group S3-10 (rolled steel – concrete joint under a normal stress of 10 N/mm^2^) (see Fig. 12). All S3-10 concrete specimens developed similar bending cracks along their lower edges, as illustrated in Fig. 12 (top right), accompanied by severe abrasion marks on the two side edges—damage patterns that were also observed, though to a lesser extent, in groups S3-1 and S3-5. The cracks along with the abrasion on the two side edges are attributed to the pronounced macro-waviness of the rolled steel, which led to surface gap in the middle. In contrast, the concrete prisms in Serie S4 showed relatively uniform abrasion marks, as also presented in Fig. 9, consistent with the smoother and more homogeneous interface geometry. Abrasion occurred on the steel prisms in series S1 – S4 as well. However, the resulting wear was too subtle to be captured clearly. For this reason, these specimens are not included in the figures. Further investigations will extend the present findings through quantitative crack analysis and microscopic characterisation of the abrasion patterns, to provide a more comprehensive understanding of the observed damage mechanisms.

#### Load-bearing behaviour

**Fig. 13:**
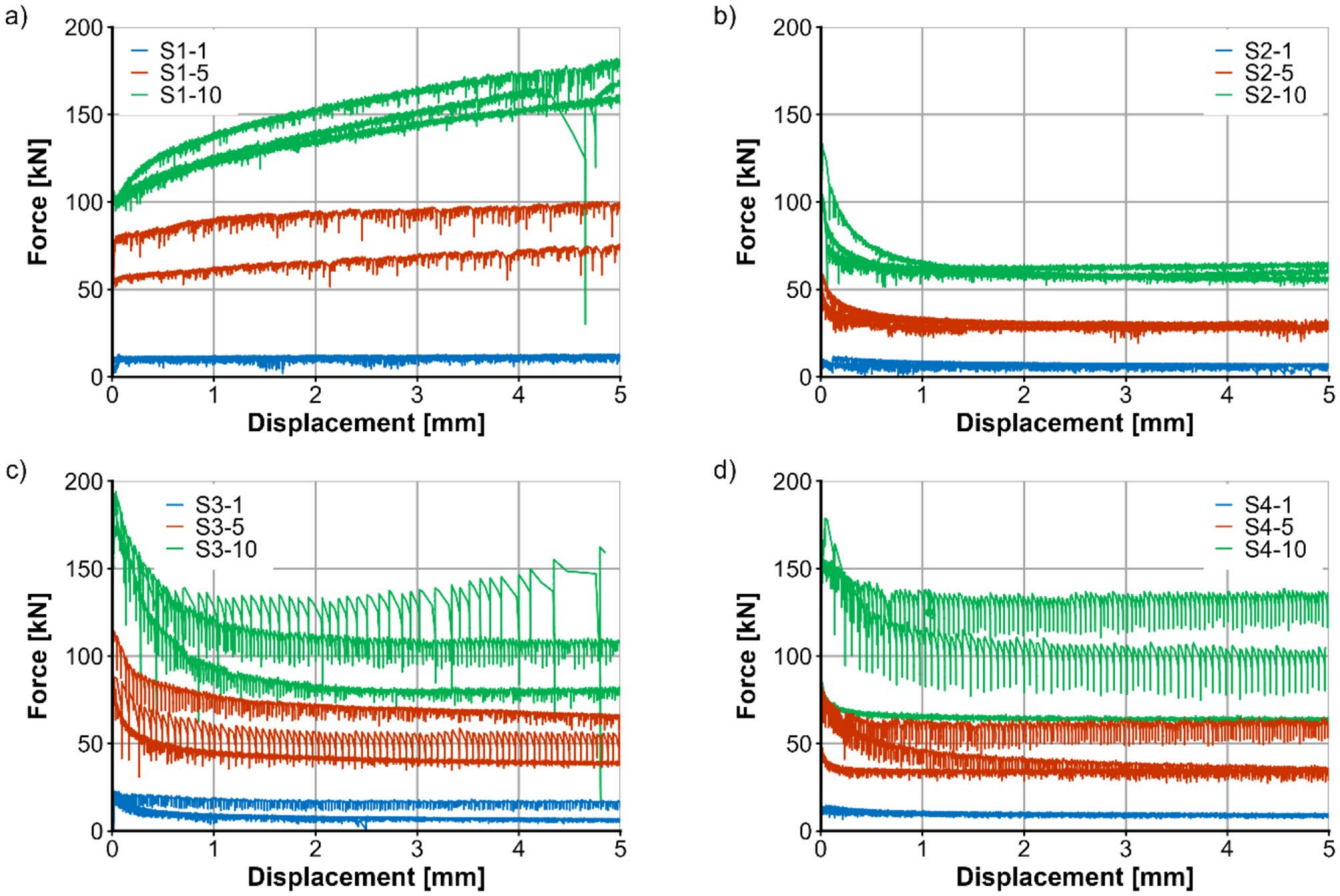
Displacement*–*force response of the push-out test for: (**a**) S1, (**b**) S2, (**c**) S3 and (**d**) S4.

The displacement–force curves obtained from the push-out tests are shown in Fig. 13. For all specimens except those in series S1, the curves exhibit the classical transition from static friction to kinetic friction (also referred to as dynamic friction), characterised by an initial force peak at very small slip followed by an exponential-type decay to a quasi-steady dynamic resistance during continued sliding. This behaviour is consistently observed across the S2 – S4 series. The markedly different behaviour observed in the S1 curves can be attributed to the pronounced mechanical interlocking generated by the macro- and micro-roughness of the rolled steel surface. Unlike the smoother interfaces in S2 – S4, where slip initiates once static friction is overcome and the response transitions rapidly into a stable kinetic regime, the S1 interface contains large asperities and macro-waviness that must be progressively sheared, crushed, or overridden during slip. As a result, the force does not drop sharply after the initial peak but instead increases or fluctuates as successive asperities engage and disengage. This repeated mobilisation and failure of interlocking features produces the irregular, non-decaying, and sometimes rising displacement–force behaviour characteristic of the series S1. In essence, the S1 response is governed not only by friction but also by shear resistance arising from geometric interlock, leading to a much more complex and non-classical frictional signature.

Additionally, Fig. 13 also revealed that the load responses are strongly governed by the surface roughness and applied normal stress. This behaviour is consistent with Coulomb’s law of friction^26^:

6 $$F=\mu \bullet (2\bullet N)$$

where *F* is the measured vertical force; *μ* is the friction coefficient; *N* is the applied normal force. The factor of two accounts for the presence of two shear interfaces in the push-out configuration. To examine differences in load-bearing capacity among the specimens, the Scheirer–Ray–Hare test^48^ was applied separately to the static and dynamic friction forces, as both variables failed the Shapiro–Wilk normality test^49^ (p-values = 0.005 and 0.001, respectively). The analysis evaluated the effects of two factors: the applied normal stress and the material combination of the joint. In this context, the static friction force is defined as the first peak load at the onset of sliding, while the dynamic friction force is calculated as the mean shear resistance within the displacement range of 2 – 5 mm. The statistical results are summarised in Table 7. The Scheirer–Ray–Hare test showed that the normal stress level is the only factor with a statistically significant effect on both the static and dynamic friction resistance (p-values < 0.0001 for both measures). In contrast, the material combination and the interaction between normal stress and material were not significant (p-values > 0.1 in all cases). Consistently, the subsequent post-hoc Wilcoxon tests^50^ revealed no significant differences among the material groups within each normal stress level. Although no significant material effect was detected, it can be observed from Fig. 13 and Table 7 that serie S1, particularly S1-1 and S1-5, exhibits consistently higher dynamic friction forces compared with the other series at the same normal-pressure level. The absence of statistical significance is plausibly explained by the lower statistical power of non-parametric tests when dealing with small sample sizes. Nevertheless, the Scheirer–Ray–Hare test remains the appropriate and robust method in this context, as the normality assumption required for parametric analysis of variance (ANOVA) was not satisfied. By operating on ranked data rather than raw values, the Scheirer–Ray–Hare test also mitigates the inflated Type-I error risk that would arise from applying ANOVA to non-normal and highly skewed data^51^. Future studies with larger replication numbers would improve statistical sensitivity and allow clearer differentiation between material combinations.

**Table 7:** Static and dynamic friction resistance of all samples obtained from push-out tests.

Sample	Static friction force			Dynamic friction force		
	Mean value [kN]	Standard deviation [kN]	Coefficient of variation [-]	Mean value [kN]	Standard deviation [kN]	Coefficient of variation [-]
S1-1	11.08	1.23	0.10	10.82	0.25	0.02
S1-5	66.62	14.33	0.22	81.04	18.18	0.22
S1-10	104.04	2.09	0.02	152.74	10.74	0.18
S2-1	10.07	1.57	0.16	6.15	1.09	0.18
S2-5	52.19	6.46	0.12	28.56	0.93	0.03
S2-10	114.28	16.92	0.15	59.05	3.26	0.06
S3-1	22.91	0.71	0.03	9.56	4.99	0.52
S3-5	96.47	15.29	0.16	51.19	13.87	0.27
S3-10	186.79	10.92	0.06	98.83	18.00	0.18
S4-1	14.03	0.63	0.04	9.13	0.20	0.02
S4-5	68.94	18.92	0.27	41.87	13.39	0.32
S4-10	139.73	48.64	0.35	94.46	31.36	0.33

In summary, no statistically significant differences in load-bearing capacity were found between the joints with different material combinations when subjected to the same normal stress. Nonetheless, the responses reveal a clear mechanical trend: as the normal stress increases, the dynamic friction resistance of S1 becomes increasingly distinct from that of the other series, reflecting its stronger interlocking behaviour. Furthermore, series S3 and S4 consistently exhibit higher static and dynamic friction forces than S2, although these differences do not reach statistical significance. These findings are particularly relevant for offshore slip-joint applications, which require high frictional resistance after installation to ensure reliable load transfer under service conditions. Although vibration-assisted assembly facilitates installation and disassembly, the frictional behaviour after installation remains decisive for structural performance. The influence of cyclic loading on the long-term evolution of friction and surface degradation under repeated environmental and operational loading should be investigated in future work.

#### Friction coefficient

**Fig. 14:**
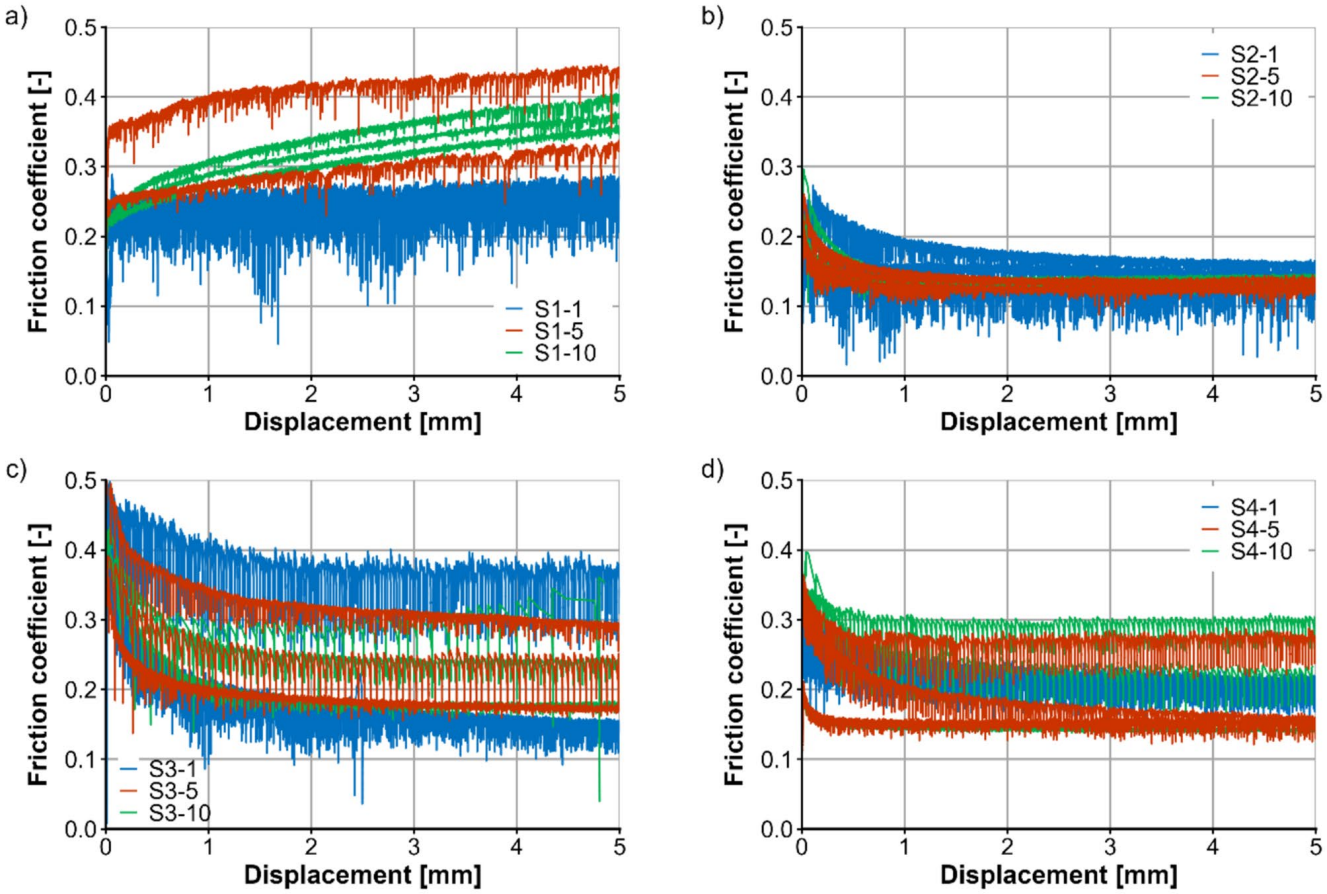
Evolution of the friction coefficient with displacement for: (**a**) S1, (**b**) S2, (**c**) S3 and (**d**) S4.

The friction coefficients *μ* for all samples, calculated using Eq. (4), are presented in Fig. 14. The observed trends are consistent with the discussion in Sect. “Load-bearing behaviour”: series S2–S4 exhibit the classical transition from static to kinetic friction, characterised by an exponential decay after the initial peak, whereas serie S1 displays an atypical increase in friction coefficient with displacement due to its pronounced interlocking behaviour. The corresponding static and dynamic friction coefficients calculated according to Table 7 are listed in Table 8. In addition to the mean values, Table 8 reports the corresponding standard deviations and coefficients of variation, providing a quantitative measure of the variability of the measured friction coefficients. To examine whether the normal stress significantly influences the friction coefficient, a Kruskal–Wallis test^52^ was applied to the static friction coefficient (which was not normally distributed), whereas a one-way ANOVA was performed on the dynamic friction coefficient (which satisfied the normality assumption). Both analyses were conducted separately within each material group. Statistical analysis showed that the normal stress did not have a significant effect on the static friction coefficient for any of the material groups. Similarly, no significant influence was observed for the dynamic friction coefficient in series S2 – S4. Only serie S1 exhibited a pressure-dependent response: specimens S1-5 and S1-10 showed significantly higher dynamic friction coefficients compared with S1-1, at significance levels of 0.05 and 0.1, respectively (p-value = 0.0457 and p-value = 0.0600). This is again attributed to the surface roughness of rolled steel, which caused a strong interlocking mechanism that intensifies as pressure increases. From an engineering perspective, the observed transition from static to dynamic friction governs the initiation of sliding and the subsequent establishment of a stable frictional resistance, which is particularly relevant for offshore slip-joint applications.

**Table 8:** Static and dynamic friction coefficients of all samples obtained from push-out tests.

Sample	Static friction coefficient			Dynamic friction coefficient		
	Mean value [-]	Standard deviation [-]	Coefficient of variation [-]	Mean value [-]	Standard deviation [-]	Coefficient of variation [-]
S1-1	0.25	0.03	0.10	0.24	0.01	0.02
S1-5	0.30	0.06	0.22	0.36	0.08	0.22
S1-10	0.23	0.01	0.02	0.34	0.02	0.07
S2-1	0.22	0.03	0.16	0.14	0.02	0.18
S2-5	0.23	0.03	0.12	0.13	0.01	0.03
S2-10	0.25	0.04	0.15	0.13	0.01	0.06
S3-1	0.51	0.02	0.03	0.21	0.11	0.52
S3-5	0.43	0.07	0.16	0.23	0.06	0.27
S3-10	0.42	0.02	0.06	0.22	0.04	0.18
S4-1	0.31	0.01	0.04	0.20	0.01	0.02
S4-5	0.31	0.08	0.27	0.19	0.06	0.32
S4-10	0.31	0.11	0.35	0.21	0.07	0.33

**Fig. 15:**
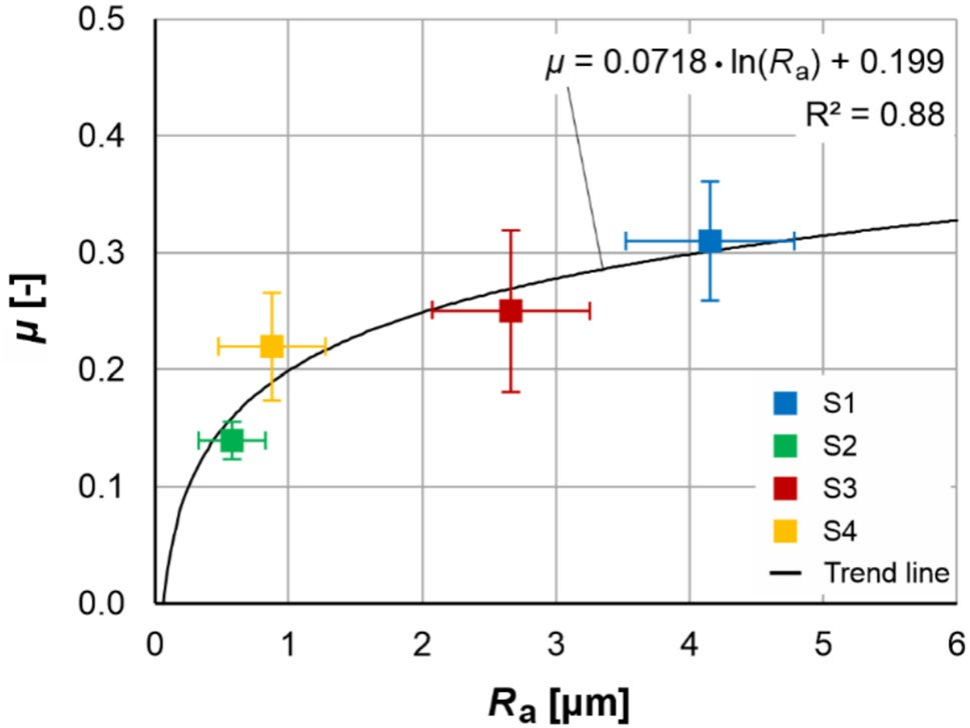
Relationship between dynamic friction coefficient and average roughness, including error bars representing the respective standard deviations.

The tendency is clear that the friction coefficient *μ* increases with increasing surface roughness ${R}_{\mathrm{a}}$. Figure 15 illustrates the relationship between the mean value of friction coefficient $\overline{\mu }$ and the mean value of the surface roughness $\overline{{R }_{\mathrm{a}}}$ of the both contact surfaces (steel-steel or steel–concrete), which is well captured by a logarithmic function, with the coefficient of determination R^2^ of 0.88.

## Summary

Based on the load-bearing behaviour and the friction–displacement characteristics of the four material configurations, the following summary can be drawn:

- S1 provides the highest shear transfer capacity due to its increasing dynamic friction and pronounced mechanical interlocking. However, this comes at the expense of substantially higher installation and disassembly forces, as well as increased wear and potential surface damage, making it less favourable for practical application.
- S2 exhibits the lowest static and dynamic friction coefficients, making it suitable for applications in which free sliding is required (e.g., expansion or sliding bearings). Nevertheless, its low friction capacity results in the lowest shear resistance among the configurations.
- S3 offers high initial and steady-state friction levels but is accompanied by pronounced stick–slip behaviour and significant concrete surface damage. This compromises long-term durability and limits its suitability for repeated or sustained slip demands.
- S4 achieves a balanced performance: its static friction coefficient is notably higher than that of S2, ensuring adequate load-bearing capacity, while its dynamic friction coefficient remains substantially lower and more stable than in S1 and S3. As a result, S4 supports moderate installation and removal forces, exhibits reduced stick–slip effects, and shows more uniform wear behaviour, making it the most practically favourable configuration overall, making it the most practically favourable configuration overall despite not achieving the maximum frictional resistance observed for S1.

The summary presented above are based on tests conducted under monotonic loading and controlled laboratory conditions. Building upon the present findings, future research will investigate the long-term evolution of friction and surface degradation under such conditions.

## Numerical simulation

### FE model

**Fig. 16:**
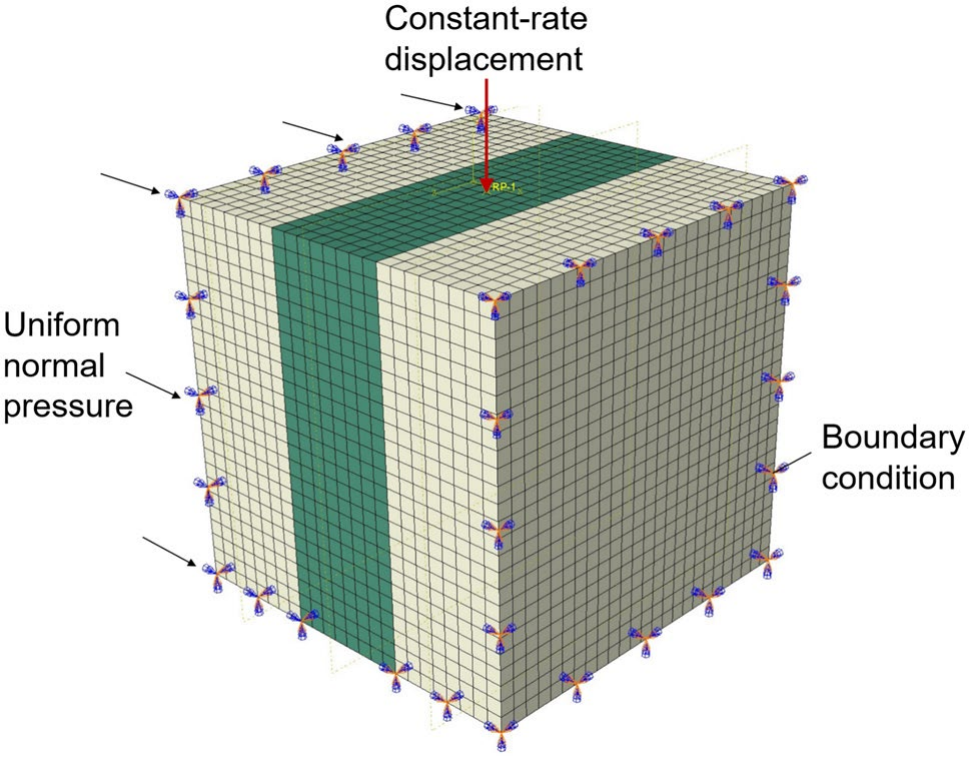
Overview of the FE model for the push-out test.

The FE simulations were performed using Abaqus/CAE 2024^53^ with the explicit solver on a workstation running Microsoft Windows 11 Pro, equipped with an Intel® Core™ Ultra 7 265H processor (16 cores) and 32 GB RAM. The computational time for a typical analysis was approximately 4–6 h. As illustrated in Fig. 16, the components were discretized using 8-node linear brick elements with reduced integration and hourglass control (C3D8R), with a uniform element size of 6 mm. A mesh sensitivity analysis was conducted on specimen S1-10 using uniform element sizes of 3, 6, and 12 mm. The influence of mesh density was evaluated by comparing the predicted reaction force responses for each mesh configuration. The differences in the peak reaction force and the reaction force at a displacement of 5 mm between the 3 and 6 mm meshes were 3.0 and 2.7%, respectively, indicating good convergence. In contrast, the corresponding differences between the 6 and 12 mm meshes exceeded 10%, suggesting that the 12 mm mesh was insufficiently refined. Considering both solution accuracy and computational efficiency, the 6 mm mesh was adopted for all subsequent analyses. Concrete and steel were both considered isotropic materials. However, it should be noted that the material was modelled as isotropic for simplicity and computational efficiency, and the model may not fully capture potential anisotropic characteristics of the materials. The concrete properties, summarised in Table 2, include a density of 2,463.7 kg/m^3^, an elastic modulus of 39,062 N/mm^2^, and a Poisson’s ratio of 0.2. The corresponding properties for steel were assumed as a density of 7,850 kg/m^3^, an elastic modulus of 210,000 N/mm^2^, and a Poisson’s ratio of 0.3. In terms of boundary conditions, the right prism was fully constrained (encastre), whereas the left prism was free to move in the normal stress direction (X-direction) to enable the transmission of the normal stress. The middle prism was unconstrained in the X-direction, while a constant velocity constraint was applied in the push-out direction (Y-direction). Furthermore, horizontal normal stresses of 1, 5 and 10 N/mm^2^ were applied to the left prism for different samples. Additionally, the contact between prisms was modelled using “surface-to-surface contact” with kinematic contact method, as it allows realistic transfer of compressive and shear stresses while permitting interface sliding and separation. The normal behaviour was modelled using “hard” contact for the pressure-overclosure relationship to prevent non-physical penetration of the contacting surfaces. In the tangential direction, frictional resistance was represented using a penalty-based friction formulation. Specifically, the friction coefficients were defined using slip-rate-dependent data following a static-to-kinetic exponential decay relationship to capture the reduction in friction during the ramp-up phase of the push-out loading rate. The decay behaviour was calibrated against the experimental response of series S2 specimens and adjusted to ensure numerical stability during the initial 10% of the analysis step. The corresponding friction coefficient values were obtained from experimental data. A limitation of the proposed contact formulation is that it may not explicitly capture the mechanical interlocking behaviour observed in the S1 specimens. Consequently, larger discrepancies between the numerical and experimental responses may be expected for this series. The nonlinear analysis was conducted using the dynamic explicit procedure without mass scaling, with automatic time incrementation governed by the explicit solver.

### Results and validation

**Fig. 17:**
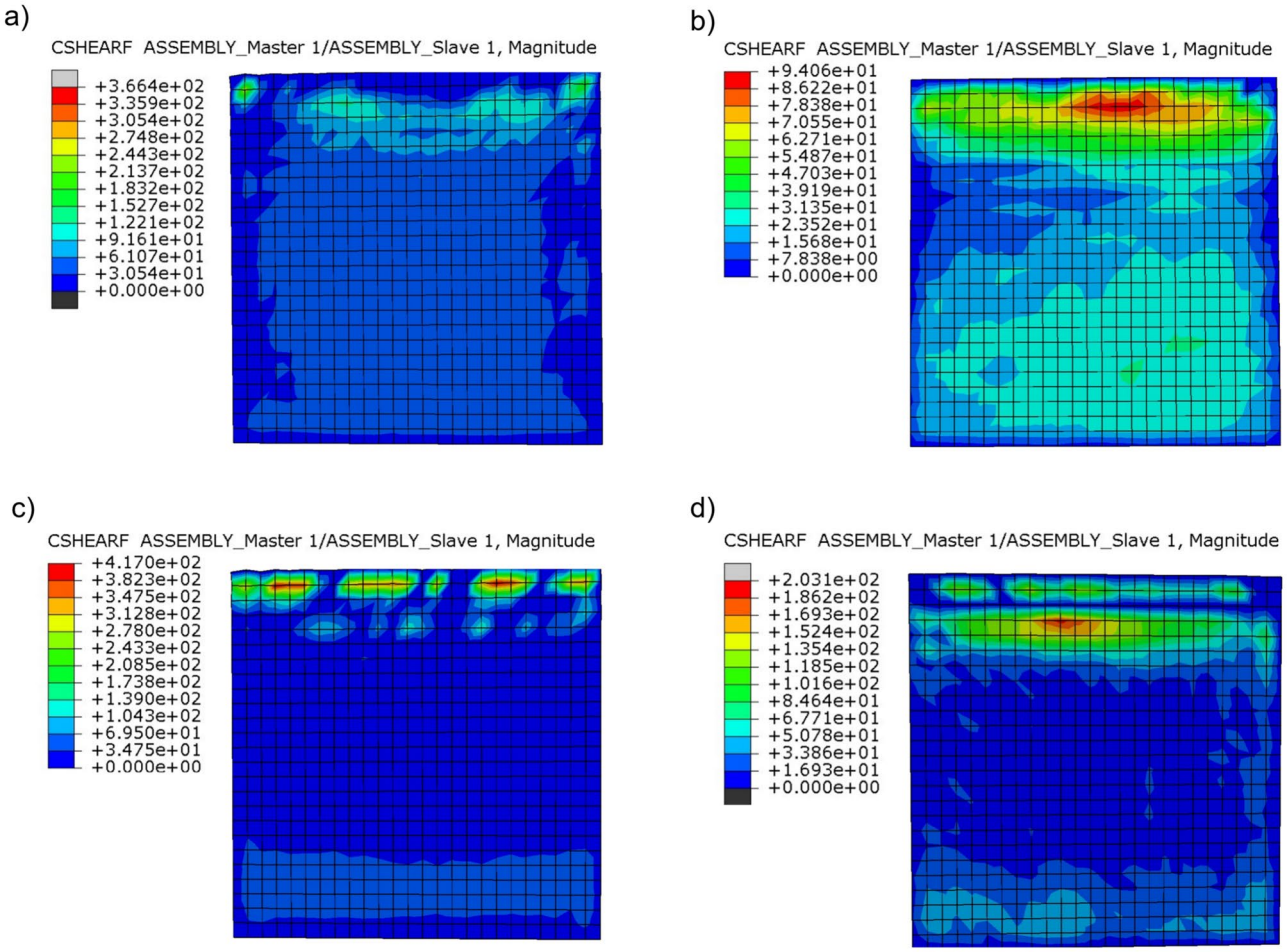
Contours of contact shear force magnitude (CSHEARF) on the left surface of the middle prism at a displacement of 2 mm for: (**a**) S1-5, (**b**) S2-5, (**c**) S3-5, and (**d**) S4-5.

Figure 17 illustrates the distribution of the contact shear force magnitude (CSHEARF) on the left surface of the middle prism. The CSHEARF values were obtained at a displacement of 2 mm under an applied normal stress of 5 N/mm^2^, at which point the shear force had reached a relatively stable state (see Fig. 13). It is notable that a stress concentration occurred at the upper part of the interface. This phenomenon can be mainly attributed to the fact that the axial load was introduced at the top boundary and progressively transferred along the interface, resulting in a shear-lag effect. Consequently, the interfacial shear stress reached its maximum near the top and gradually decayed with depth. This stress concentration may also contribute to the cracking observed in S3-10 (Fig. 12), in addition to the macro-waviness identified through 3D scanning. In addition, as shown in Fig. 17, the sample with a lower friction coefficient (i.e., S2-5 and S4-5 compared with S1-5 and S3-5, respectively) exhibited a lower CSHEARF peak near the top and a more uniform CSHEARF distribution along the interface. This behaviour occurred because a lower friction coefficient reduced the maximum shear traction that could be sustained before sliding began, allowing slip to initiate over a larger portion of the interface and resulting in a more uniform shear transfer.

**Fig. 18:**
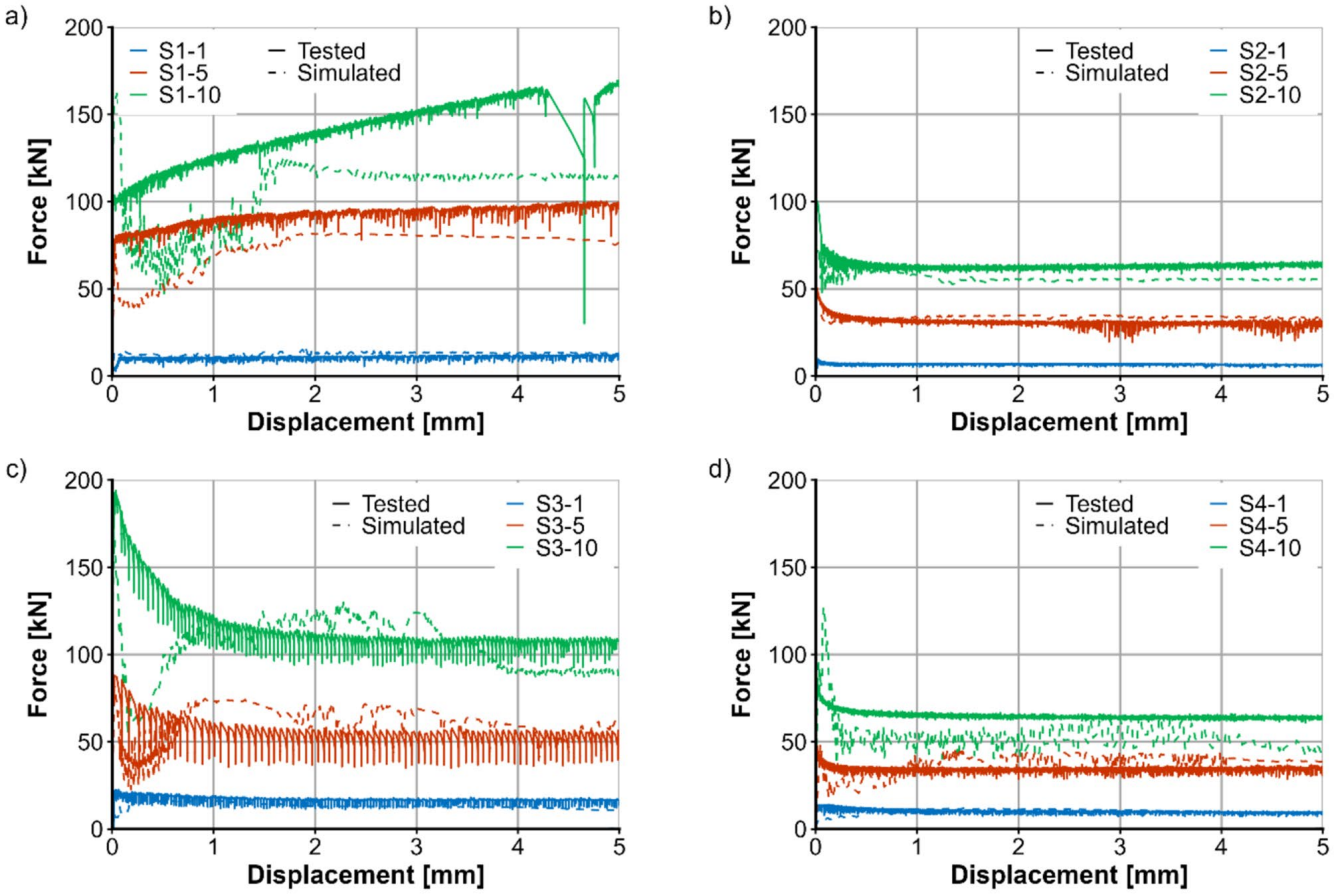
Comparison of simulated and experimental displacement-force curves: (**a**) S1, (**b**) S2, (**c**) S3 and (**d**) S4.

Figure 18 compares the simulated and experimental displacement-force curves for all specimens. The experimental results shown are mean curves derived from three replicate tests (cf. Fig. 13). Noticeable fluctuations appear in several simulated curves, especially for series S3 and S4 and for cases with higher applied normal stress. These fluctuations can be attributed, on one hand, to the differing material behaviours of steel and concrete, which lead to an uneven composite response. On the other hand, it is more likely caused by the numerical instabilities inherent in the dynamic explicit analysis procedure. Minor oscillations may also result from intermittent stick–slip transitions at the interface between prisms. Nevertheless, the kinetic-to-internal energy ratio remained below 5%, confirming that the simulations predominantly exhibited quasi-static behaviour. Additionally, it should be acknowledged that the agreement between the simulated and experimental curves in Serie S1 is less pronounced. As discussed in Sect. 3.6, during testing, an interlocking effect occurred during sliding between the rolled steel surfaces due to their pronounced surface roughness. This caused the contact interaction in Serie S1 to deviate from the typical frictional behaviour described by the static-kinetic exponential decay law. Instead of a smooth reduction in friction with the onset of sliding, local mechanical interlocking between asperities on the rolled steel surfaces generated irregular resistance and a gradual increase in frictional force (slide hardening), which was not captured by the numerical model.

Static and dynamic friction forces are critical parameters influencing the entire life cycle of slip joints in offshore substructures. The static friction force governs the load-bearing capacity and slip resistance during service, while the dynamic friction force controls the resistance encountered during installation and sliding. Both parameters also affect the effort required for disassembly at the end of the service life. Therefore, reliable prediction of static and dynamic friction is essential for accurate structural design, safe installation, and realistic assessment of long-term performance. To ensure that the numerical model captures these key behaviours, the simulated friction forces were validated against experimental results. Table 9 presents the tested and predicted values, where the static friction force corresponds to the first peak at the onset of sliding, and the dynamic friction force is obtained as the mean value within the displacement range of 2 – 5 mm. The relative errors remain below 30% for all cases except S1-10. As explained earlier, the numerical model does not reproduce the behaviour of serie S1 with the same level of accuracy as the other samples. Despite these limitations, the FE model generally demonstrates good agreement with the push-out test results, as evidenced by the validation metrics presented in Table 10, including the coefficient of determination (R^2^), mean absolute percentage error (MAPE), and root mean square error (RMSE). These results indicate that the model is capable of reasonably predicting the load-bearing behaviour of slip joints and has strong potential for application in future numerical investigations and design assessments.

**Table 9:** Comparison of tested and predicted static and dynamic friction forces.

Sample	Static friction force			Dynamic friction force		
	Tested [kN]	Predicted [kN]	Relative error [%]	Tested [kN]	Predicted [kN]	Relative error [%]
S1-1	10.94	12.42	13.53	10.58	13.29	25.61
S1-5	80.51	70.05	12.99	93.49	79.79	14.65
S1-10	102.01	161.16	57.98	144.93	115.03	20.63
S2-1	9.56	8.25	13.70	6.43	6.77	5.29
S2-5	52.03	37.74	27.47	29.17	33.89	16.18
S2-10	100.49	72.55	27.80	62.68	55.34	11.71
S3-1	20.03	16.91	15.58	15.27	12.37	18.99
S3-5	86.38	82.26	4.77	47.49	60.53	27.46
S3-10	193.07	166.00	14.02	103.86	104.46	0.58
S4-1	13.16	11.63	11.63	9.32	10.18	9.23
S4-5	47.51	47.78	0.57	33.37	39.93	19.66
S4-10	85.21	95.69	12.30	63.70	53.88	15.42

**Table 10:** Performance of the FE model in predicting static and dynamic friction forces.

Response	MAPE (%)	RMSE	R^2^
Static friction force	17.69	21.35	0.83
Dynamic friction force	15.45	11.12	0.93
Combined dataset	16.57	17.02	0.87

In summary, based on the simulated results, S2 is recommended for use in steel–steel slip joints, while S4 is more suitable for steel–concrete slip joints. Although S1 provides higher frictional resistance, which can enhance load-bearing capacity during service, its irregular surface roughness leads to less predictable frictional behaviour. Furthermore, the pronounced interlocking effect in S1 may induce shear hotspots and stress concentration at the interface, which could accelerate wear or initiate local damage over long-term service. Similarly, when compared with S4, the S3 configuration exhibited a more pronounced stress concentration at the upper part of the interface, as shown in Fig. 17. Such localised stress intensification can generate microcracks within the surrounding concrete, particularly near the joint entrance. Consequently, to ensure better structural integrity and long-term durability, S4 is considered more favourable for steel–concrete slip joints.

## Conclusions and outlook

This study proposes innovative steel–steel and steel–concrete slip joint for modular jacket offshore substructure. The investigation compares contact surface morphology, joint fitting accuracy, friction coefficients and load-bearing behaviour among rolled/ground steel–rolled/ground steel and rolled/ground steel–concrete joint configurations. Furthermore, a numerical model is developed to simulate the push-out tests and predict the load-bearing capacity of the joints. The main findings are as follows:

- The 3D scanning method provided roughness measurements comparable to the conventional tactile method while enabling full-surface characterisation, including both micro- and macro-roughness, and quantification of joint fitting accuracy.
- Unlike the S2, S3 and S4 joints, which exhibited a conventional static-to-kinetic friction transition, the S1 joints showed pronounced mechanical interlocking, resulting in increasing friction resistance and friction coefficient with displacement. This effect became more pronounced with increasing normal stress.
- Only the series S1 exhibited a normal-pressure-dependent friction coefficient, attributed to the pronounced asperities of the rolled-steel surface. Overall, surface roughness was identified as the dominant parameter governing frictional behaviour, with the friction coefficient following a logarithmic relationship with average roughness.
- The FE model captured the observed shear-lag behaviour, with interfacial shear stresses concentrated near the loaded end and decreasing with depth. Good agreement with the experimental results was achieved overall, although larger deviations were observed for S1-10 due to the complex interlocking behaviour not explicitly represented in the contact formulation.

This study demonstrates the strong potential of employing steel–steel and steel–concrete slip joints in modular jacket-type offshore substructures. For steel–steel slip joints, the ground steel–ground steel configuration (series S2) exhibited more predictable frictional behaviour, whereas the rolled steel–rolled steel configuration provided higher frictional resistance. For steel–concrete slip joints, the ground steel–concrete configuration (series S4) showed reduced stick–slip behaviour, moderate assembly forces, and more uniform wear characteristics. These findings suggest that S4 may offer a favourable balance between frictional performance and assembly requirements. However, further testing is required to confirm the observed differences between S3 and S4.

A few limitations of this study should be acknowledged. The investigation was conducted using quasi-static push-out tests under controlled laboratory conditions and therefore does not fully represent the complex loading and environmental conditions encountered by offshore structures, such as cyclic loading, environmental exposure, and installation-induced dynamic effects. In addition, long-term surface wear, friction degradation, and scale effects were not considered. Therefore, the findings should be regarded as a fundamental investigation of interface behaviour.

Building on the findings of the present work, further experimental and numerical investigations will be carried out on representative slip joint configurations with regard to installation process, load-bearing capacity under compression and tensile load (see Fig. 2), to validate and refine the observed behaviours. Ultimately, the aim is to develop innovative slip joint connections that are capable of transferring high axial compressive loads and, above all, high axial tensile loads, thus enabling modular mega-substructures and significantly expanding the design space for offshore megastructures.

## Data Availability

The datasets generated and/or analysed during the current study, including experimental measurements and numerical simulation results, are available from the corresponding author on reasonable request.

## Abbreviations

$${a}_{\mathrm{j}}$$: Surface distance
*c*: Shape parameter in Burr type XII distribution
*d**f*: Degree of freedom
${f}_{\mathrm{c}\mathrm{m}}$: Mean value of concrete compressive strength
$${l}_{\mathrm{e}}$$: Evaluation length
*k*: Tail-shape parameter in Burr type XII distribution
*z(x)*: Ordinate values of the surface profile over evaluation length
*z(x,y)*: Ordinate values of the surface profile over evaluation area
*A*: Evaluation area
${A}_{\mathrm{g}}$: Sum of all areas ${A}_{\mathrm{o}\mathrm{i}}$ and ${A}_{\mathrm{u}\mathrm{i}}$ of an evaluation length
${A}_{\mathrm{o}\mathrm{i}}$: Areas above the abscissa
${A}_{\mathrm{u}\mathrm{i}}$: Areas below the abscissa
*ANOVA*: Analysis of variance
*CSHEARF*: Contours of contact shear force magnitude
${E}_{\mathrm{c}\mathrm{m}}$: Mean value of concrete E-modulus
*F*: Vertical load
*FE*: Finite element
*HPC*: High-performance concrete
*MAPE*: Mean absolute percentage errors
*N*: Normal load
${R}_{\mathrm{a}}$: Arithmetic mean height
*R*^*2*^: Coefficient of determination
${S}_{\mathrm{a}}$: Arithmetic mean height
*T*: Likelihood-ratio test statistic
*wt.%*: Weight percent
*μ*: Friction coefficient
${\rho}_{\mathrm{c}\mathrm{m}}$: Mean value of concrete bulk density
$${\sigma}_{\mathrm{n}}$$: Normal stress
*λ*: Scale parameter in Burr type XII distribution
